# Supplementary material for: Differentiating the learning styles of college students in different disciplines in a college English blended learning setting
Source: PLoS One. 2021 May 20;16(5):e0251545. doi: 10.1371/journal.pone.0251545 (PMC8139239; doi:10.1371/journal.pone.0251545)
Supplement: S1 File — (DOCX) [file pone.0251545.s001.docx]

**S1 File.**

**S1 Fig. The adapted Felder-Silverman learning style model.**


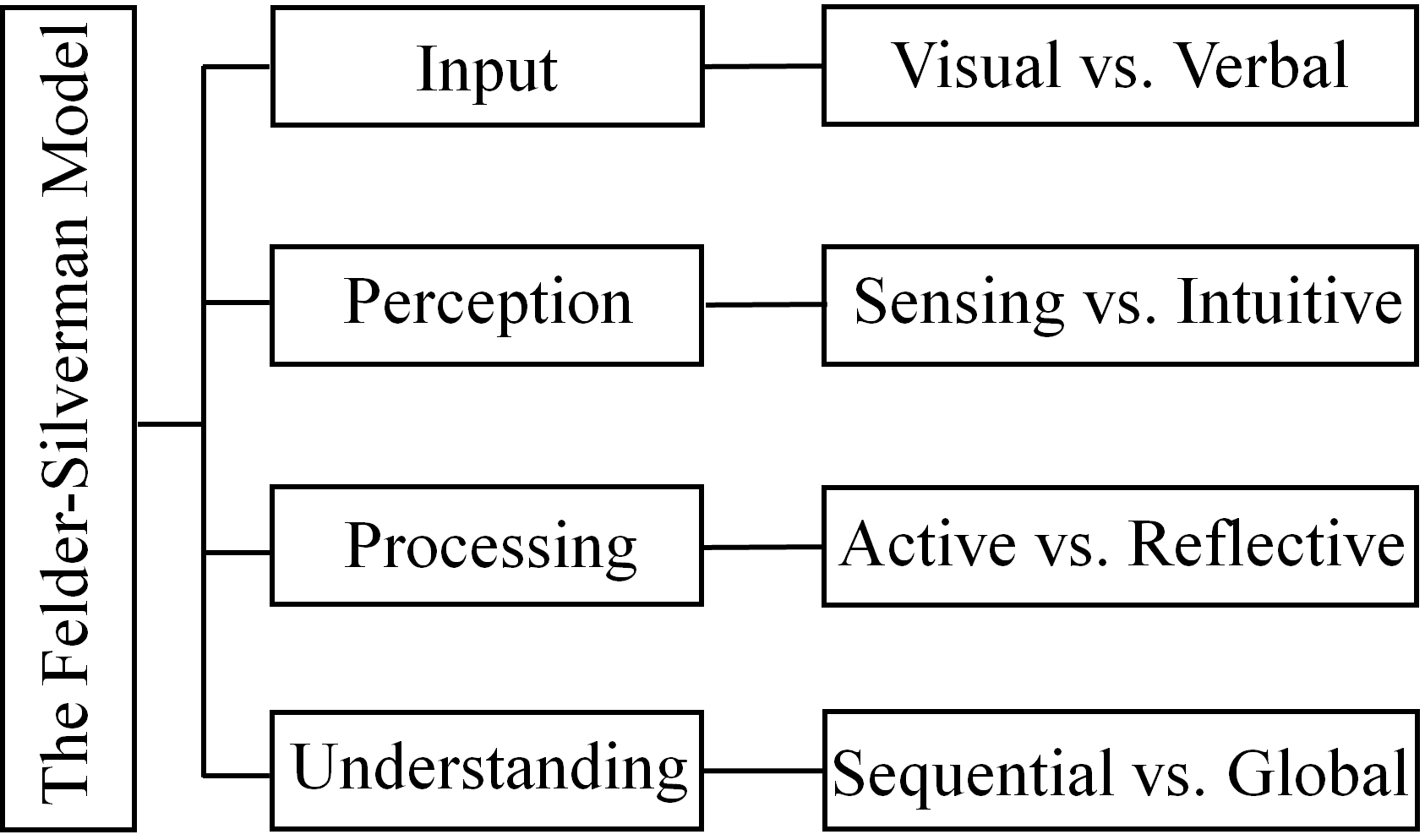


**Fig 1.** The adapted Felder–Silverman learning style model.

*Note.* This model specifies the four dimensions of the construct of learning style: visual/verbal, sensing/intuitive, active/reflective, and sequential/global. These four dimensions correspond to four psychological processes: input, perception, processing, and understanding.

**S2 Fig. The mechanism underlying the support vector machine.**


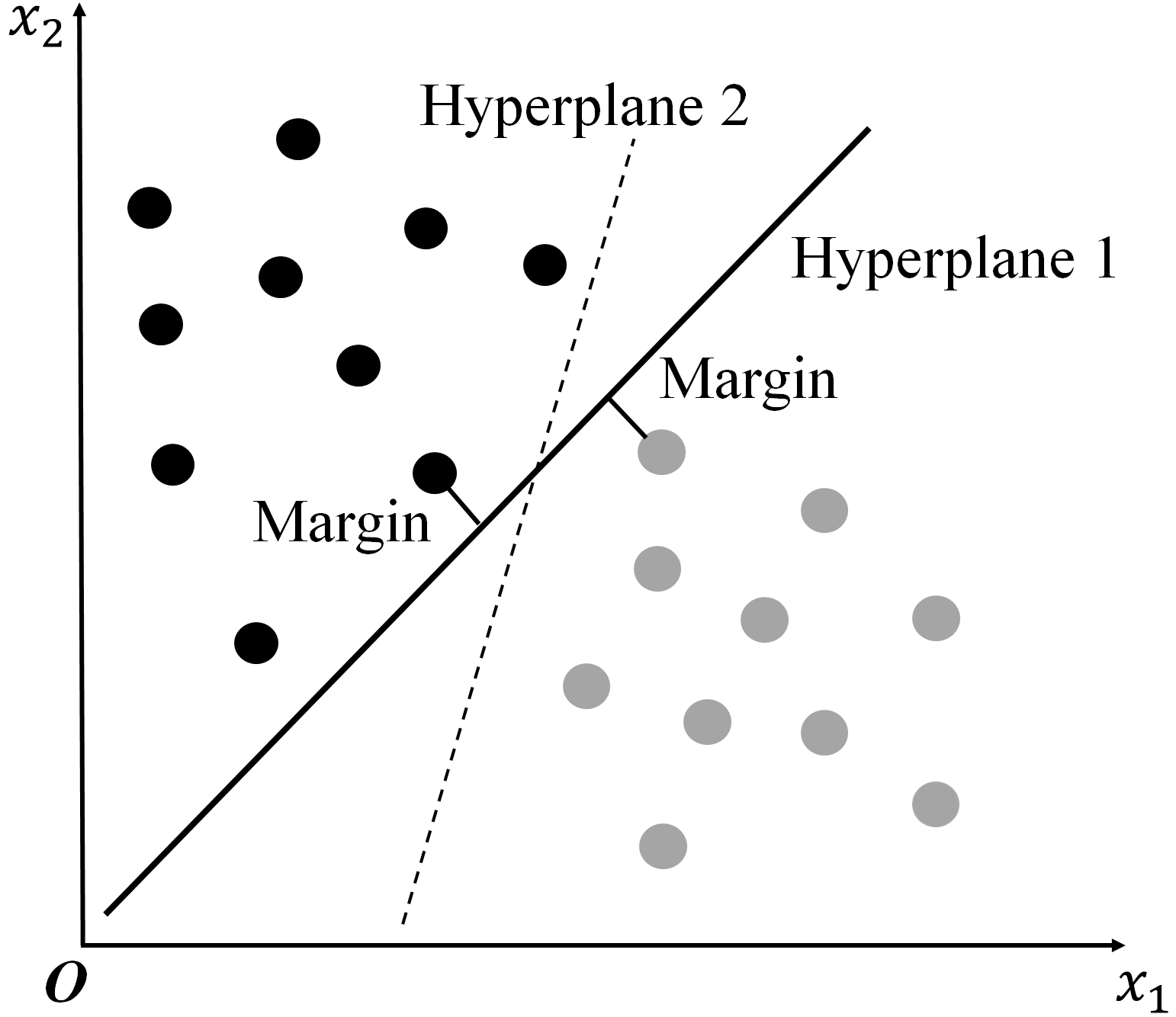


**Fig 2.** The mechanism underlying the support vector machine.

*Note.* Hyperplanes 1 and 2 are two regression lines that divide the data into two groups. Hyperplane 1 is considered the best fitting line because it maximizes the distance between the two groups.

**S3 Fig.** **Experimental process and working mechanism of SVM and SVM-RFE.**


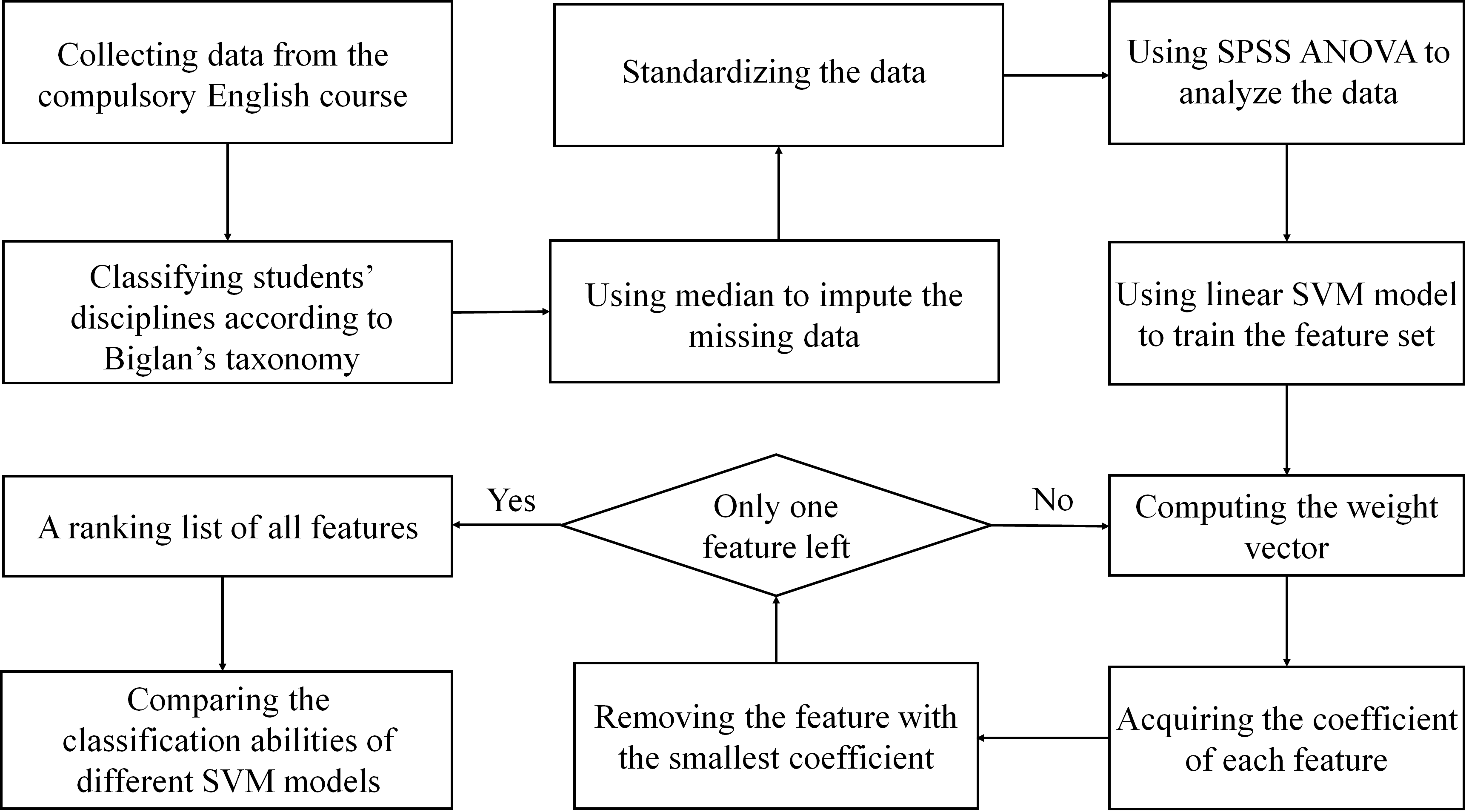


**Fig 3.** Experimental process and working mechanism of SVM and SVM-RFE.

**S1 Table. Description of a confusion matrix.**

**Table 1. Description of a confusion matrix.**

|  | **Positive (Predicted)** | **Negative (Predicted)** |
| --- | --- | --- |
| **Positive (Actual)** | True Positive (TP) | False Negative (FN) |
| **Negative (Actual)** | False Positive (FP) | True Negative (TN) |

*Note.* Positive: Observation is positive (e.g., the students belong to this discipline); Negative: Observation is negative (e.g., the students do not belong to this discipline); True Positive (TP): Observation is positive and is predicted to be positive; False Negative (FN): Observation is positive but is predicted to be negative; True Negative (TN): Observation is negative and is predicted to be negative; False Positive (FP): Observation is negative but is predicted to be positive.

**S2 Table.** **Results produced by SVM and SVM-RFE.**

**Table 2. Results produced by SVM and SVM-RFE.**

| **Model** | **Algorithm** | **ACC** | **SPE** | **SEN** | **AUC** | **F-measure** | **Top 20 features** |
| --- | --- | --- | --- | --- | --- | --- | --- |
| **H-S** | SVM | 66.67% | 69.32% | 63.38% | 66.35% | 66.67% | 1, 2, 4, 5, 7, 8, 9, 10, 11, 14, 21, 22, 23, 26, 27, 29, 31, 32, 35, 39 |
|  | SVM-RFE | 87.30% | 88.18% | 86.20% | 87.19% | 87.30% |  |
| **A-P** | SVM | 64.52% | 77.78% | 55.08% | 64.93% | 64.52% | 3, 4, 5, 6, 7, 11, 15, 17, 18, 19, 21, 30, 31, 32, 33, 35, 39, 42, 43, 44 |
|  | SVM-RFE | 73.44% | 86.55% | 79.28% | 75.09% | 74.44% |  |
| **HA-HP** | SVM | 69.32% | 80.00% | 67.95% | 73.97% | 69.32% | 1, 4, 5, 6, 7, 8, 10, 12, 13, 14, 19, 21, 26, 28, 29, 31, 34, 39, 40, 44 |
|  | SVM-RFE | 82.59% | 82.30% | 78.26% | 89.13% | 79.59% |  |
| **HA-SA** | SVM | 68.47% | 63.64% | 70.51% | 67.07% | 68.47% | 1, 2, 4, 7, 8, 9, 10, 11, 14, 18, 21, 26, 27, 29, 32, 35, 37, 38, 39, 41 |
|  | SVM-RFE | 77.74% | 75.00% | 68.22% | 78.11% | 77.74% |  |
| **HA-SP** | SVM | 68.97% | 73.68% | 66.67% | 70.18% | 68.97% | 1, 2, 7, 9, 10, 11, 12, 14, 19, 20, 21, 22, 23, 27, 31, 32, 34, 35, 37, 39 |
|  | SVM-RFE | 76.09% | 74.00% | 67.70% | 77.35% | 69.09% |  |
| **HP-SA** | SVM | 55.16% | 56.52% | 58.00% | 57.76% | 56.16% | 1, 2, 4, 5, 6, 7, 9, 10, 12, 15, 16, 21, 26, 27, 28, 29, 31, 33, 39, 44 |
|  | SVM-RFE | 74.63% | 72.00% | 70.04% | 75.02% | 69.63% |  |
| **HP-SP** | SVM | 58.33% | 57.90% | 60.00% | 58.95% | 58.33% | 1, 2, 5, 7, 8, 12, 13, 15, 16, 18, 19, 21, 23, 27, 29, 32, 33, 34, 35, 40 |
|  | SVM-RFE | 70.83% | 75.00% | 68.00% | 72.50% | 70.83% |  |
| **SA-SP** | SVM | 60.00% | 63.16% | 56.25% | 59.70% | 60.00% | 3, 4, 5, 7, 8, 9, 11, 13, 15, 18, 19, 21, 27, 31, 32, 35, 37, 39, 41, 44 |
|  | SVM-RFE | 70.46% | 75.00% | 76.25% | 71.28% | 71.46% |  |

*Note*: Indicators in the upper row belong to original SVM models, while those in the lower row belong to the models with 20 features.

**S3 Table. Question descriptions of the top 20 features in the HA-HP model.**

**Table 3. Question descriptions of the top 20 features in the HA-HP model.**

| **Question**  **Number** | **Question** | **Answer Option** |
| --- | --- | --- |
| **1** | I understand something better after I | A. try it out. |
|  |  | B. think it through. |
| **4** | I tend to | A. understand the details of a subject but may be fuzzy about its overall structure. |
|  |  | B. understand the overall structure but may be fuzzy about the details. |
| **5** | When I am learning something new, it helps me to | A. talk about it. |
|  |  | B. think about it. |
| **6** | If I were a teacher, I would rather teach a course | A. that addresses facts and real-life situations. |
|  |  | B. that addresses ideas and theories. |
| **7** | I prefer to obtain new information from | A. pictures, diagrams, graphs, or maps. |
|  |  | B. written directions or verbal information. |
| **8** | Once I understand | A. all the parts, I understand the whole thing. |
|  |  | B. the whole thing, I see how the parts fit. |
| **10** | I find it easier | A. to learn facts. |
|  |  | B. to learn concepts. |
| **12** | When I solve math problems | A. I usually work my way to the solutions one step at a time. |
|  |  | B. I often just see the solutions but then have to struggle to figure out the steps to get to them. |
| **13** | In the classes I have taken | A. I usually got to know many students. |
|  |  | B. I rarely got to know many students. |
| **14** | In reading nonfiction, I prefer | A. something that teaches me new facts or tells me how to do something. |
|  |  | B. something that gives me new ideas to think about. |
| **19** | I remember best | A. what I see. |
|  |  | B. what I hear. |
| **21** | I prefer to study | A. in a study group. |
|  |  | B. alone. |
| **26** | When I am reading for enjoyment, I like writers to | A. clearly say what they mean. |
|  |  | B. say things in creative, interesting ways. |
| **28** | When considering a body of information, I am more likely to | A. focus on the details and miss the big picture. |
|  |  | B. try to understand the big picture before getting into the details. |
| **29** | I more easily remember | A. something I have done. |
|  |  | B. something I have thought a lot about. |
| **31** | When someone is showing me data, I prefer | A. charts or graphs. |
|  |  | B. text summarizing the results. |
| **34** | I consider it higher praise to call someone | A. sensible. |
|  |  | B. imaginative. |
| **39** | For entertainment, I would rather | A. watch television. |
|  |  | B. read a book. |
| **40** | Some teachers start their lectures with an outline of what they will cover. Such outlines are | A. somewhat helpful to me. |
|  |  | B. very helpful to me. |
| **44** | When solving problems in a group, I would be more likely to | A. think of the steps in the solution process. |
|  |  | B. think of possible consequences or applications of the solution in a wide range of areas. |

*Note.* Question descriptions and answer options were openly accessed online from the ILS (URL: https://www.webtools.ncsu.edu/learningstyles/).
