# Supplementary material for: Differentiating the learning styles of college students in different disciplines in a college English blended learning setting
Source: PLoS One. 2021 May 20;16(5):e0251545. doi: 10.1371/journal.pone.0251545 (PMC8139239; doi:10.1371/journal.pone.0251545)
Supplement: S2 File — (DOCX) [file pone.0251545.s002.docx]

**S****2 File. Informed consent** **for participants.**

**Informed Consent**

Thanks for your participation in this test. Before you answer the questions, there are a few points you need to know

1. This test intends to provide an estimation of your **learning style**, serving as an important part of your English courses this semester.
2. Please finish this test carefully. You may only choose one answer for each question, and you must answer all questions before you can submit the form. If both answers to a question seem to apply to you, choose the one that applies **more frequently throughout all your courses**.
3. Your personal information would be used for **research purposes only**.

If you agree with the above, please tick in the box here:
