# Supplementary material for: Differentiating the learning styles of college students in different disciplines in a college English blended learning setting
Source: PLoS One. 2021 May 20;16(5):e0251545. doi: 10.1371/journal.pone.0251545 (PMC8139239; doi:10.1371/journal.pone.0251545)
Supplement: Attachment — Submitted filename: Authors Responses to Reviewers Comments.docx [file pone.0251545.s004.docx]

**Authors’ Responses to Reviewers’ Comments**

To,

The Editor and the Esteemed Reviewers,

PLOS ONE,

Sub: Submission of revised manuscript (Manuscript ID: PONE-D-20-14494)

Dear Sirs/Madams,

We would like to express our sincere thanks for reviewing our manuscript entitled “Differentiating the learning styles of EFL students with different disciplines in a blended learning setting: Insights from a machine learning-based algorithm” and providing us an opportunity to make corrections in it. As per your valuable comments, the manuscript has been revised and all the amendments suggested by the reviewers have been incorporated.

Considering the heavy workload in revising the manuscript and other engagement, another author has joined the project in the revising process. Therefore, we would like to submit an authorship change form here.

All the authors have reviewed and approved this version of the manuscript and declared no conflicts of interest. This manuscript has not been submitted to any other journal or published before.

Once again, we are thankful to all of the reviewers for promote the quality of our manuscript!

With best regards,

Authors

**Responses to Reviewers’ Comments**

Table of Contents

[Responses to the Academic Editor	2](#_Toc50237609)

[Requirement 1	2](#_Toc50237610)

[Requirement 2	2](#_Toc50237611)

[Requirement 3	4](#_Toc50237612)

[Requirement 4	4](#_Toc50237613)

[Responses to Reviewer #1	6](#_Toc50237614)

[Comment 1	6](#_Toc50237615)

[Comment 2	6](#_Toc50237616)

[Comment 3a	6](#_Toc50237617)

[Comment 3b	12](#_Toc50237618)

[Comment 3c	13](#_Toc50237619)

[Comment 4	17](#_Toc50237620)

[Comment 5	18](#_Toc50237621)

[Comment 6	18](#_Toc50237622)

[Responses to Reviewer #2	20](#_Toc50237623)

[Comment 1	20](#_Toc50237624)

[Comment 2	20](#_Toc50237625)

[Comment 3	20](#_Toc50237626)

[Comment 4	25](#_Toc50237627)

[Comment 5	29](#_Toc50237628)

[Comment 6	30](#_Toc50237629)

[Comment 7	33](#_Toc50237630)

[Comment 8	34](#_Toc50237631)

[Comment 9	34](#_Toc50237632)

**Responses to the Academic Editor**

**Requirement 1**

Please ensure that your manuscript meets PLOS ONE's style requirements, including those for file naming.

**Response to requirement 1**

Thanks to the editor for the kind remainder. Necessary changes have been made according to the PLOS ONE's style requirements. In addition, we have revised the name of the file as “Revised Manuscript with Track Changes”, and have inspected the whole manuscript.

**Requirement 2**

Please upload a copy of the questionnaire used in the study as a supplemental file or provide a URL where it can be accessed. Additionally, although you state that faculty gave permission for their students to complete the questionnaire, please state in both your Ethics Statement and your Methods section whether informed consent was given by the participants who completed the questionnaire or whether the need for consent was waived by your ethics approval board. Please state what type of consent was given (i.e., written, verbal, etc.).

**Response to requirement 2**

Thanks to the editor for this recommendation. The URL of the ILS questionnaire we used in this research is provided in Lines 301-303, and is listed below in blue font for reference:

Fourth, the ILS is a free instrument that can be openly accessed online (URL: https://www.webtools.ncsu.edu/learningstyles/) and has been widely used in the research context [69-70].

We felt sorry that in the original manuscript, we did not include the Ethic Statements and type of consent provided by the participants. In the revised version, we have added this important missing information in the Ethics Statement and the Methods sections. The ethic statements were added in the section of “Materials and methods”. So is the type of consent given by the students. Both of these modifications are provided below for reference:

(Lines 323-330) Before the questionnaires were distributed, the researchers involved in this study contacted faculty members from various departments and requested their help. After permission was given, the printed questionnaires were administered to students under the supervision of their teachers at the end of their English language course. The students were informed of the purpose and importance of the study and asked to carefully complete the questionnaires. The students were also assured that their personal information would be used for research purposes only. All students provided written informed consent (see Supplementary Material).

……

(Lines 733-741) **Ethical Statements**

The questionnaires were administered in a single session by specialized staff who collaborated in the investigation. Participants completed all the questionnaires individually. The procedures followed in the study were in accordance with the ethical standards of the Helsinki Declaration and were approved by the school administration team and granting agency. All participants signed written informed consent to authorize their participation in this research. When filling in the informed consent form, each participant was provided a gift (a pen) in gratitude for their contribution and participation.

…….

**Supplementary Material**: Informed consent for participants.

**Informed Consent**

Thanks for your participation in this test. Before you answer the questions, there are a few points you need to know

1. This test intends to provide an estimation of your **learning style**, serving as an important part of your English courses this semester.
2. Please finish this test carefully. You may only choose one answer for each question, and you must answer all questions before you can submit the form. If both answers to a question seem to apply to you, choose the one that applies **more frequently throughout all your courses**.
3. Your personal information would be used for **research purposes only**.

If you agree with the above, please tick in the box here:

**Requirement 3**

Please ensure that you include a title page within your main document. We do appreciate that you have a title page document uploaded as a separate file, however, as per our author guidelines (http://journals.plos.org/plosone/s/submission-guidelines#loc-title-page) we do require this to be part of the manuscript file itself and not uploaded separately.

Could you therefore please include the title page into the beginning of your manuscript file itself, listing all authors and affiliations.

**Response to requirement 3**

Many thanks to the editor for this invaluable suggestion. We have now revised the manuscript to include the title page at the beginning, where all authors and affiliations are listed.

**Requirement 4**

Please include captions for your Supporting Information files at the end of your manuscript, and update any in-text citations to match accordingly. Please see our Supporting Information guidelines for more information: [http://journals.plos.org/plosone/s/supporting-information](about:blank).

**Response to requirement 4**

The suggestion from the editor is greatly appreciated. We have newly added a section for Supporting Information where all the captions included in the manuscript are indexed and have updated the in-text citations to match accordingly.

## Responses to Reviewer #1

### Comment 1

This study aims to introduce an innovative machine learning algorithm known as support vector machine (SVM) and specify the impact of students’ disciplinary background on their learning styles in EFL blended learning, which is interesting and investigation worthy. The method is inspiring and well-designed.

**Response to comment 1**

Many thanks to the reviewer for this positive comment.

### Comment 2

The authors may further improve this paper in the following points.

**Response to comment 2**

Thanks for this valuable suggestion. In the following parts, we will present our point-by-point responses to your valuable comments.

### Comment 3a

There is no literature review in this paper, leading to the insufficient establishment of theoretical framework of this paper. Before leading in the research method, a few questions from three main aspects are supposed to be answered based on the literature. (a) “what is the definition of learning style?” “What are the major learning styles of EFL learning/blended learning/EFL blended learning as shown in the literature?” “From which aspects could learning styles influence EFL learning/blended learning/EFL blended learning?”

**Response to comment 3a**

We are extremely appreciative of the reviewer’s comment, which give us a clear sense of what we missed and should do when revising it. We apologize for our negligence, which has not only led to the absence of detailed interpretation of the research object, “learning style”, but also resulted in the insufficient literature-based evidence in our research. Learning style, as a vital topic of education, has been constructed by many influential scholars. During the revision, we have avoided any misunderstanding by clearly illustrating the definitions of this term. In addition, the academic discipline which is regarded as a significant influencing factor of learning styles has also been carefully examined by a great many researchers, which provides a solid foundation for our research. In the revised manuscript, we have carefully included these inspiring efforts.

According to the reviewer’s valuable suggestion, we took the effort to rewrite basically all of the introduction section in the manuscript, and made substantial revisions to the rest of the manuscript wherever this is necessary, which include the different aspects suggested by the reviewer. The introduction which includes literature review is now listed below for the reviewer’s reference, and the modifications are highlighted in blue font here and in the revised manuscript.

In particular, the first paragraph serves as the research background, giving a basic introduction of the term “learning style”:

(Lines 55-60) Learning style, as an integral and vital part of students’ learning process, has always been a long-lasting and hot-debated topic in the field of education and pedagogy. Originally developed from the field of psychology, psychological classification, and cognitive research several decades ago [1], the term “learning style” is generally defined as the learner’s innate and individualized preference for ways of participation in learning practice [2].

The second paragraph, following upon that, provides the definitions, interpretations and constructions of this term proposed by some influential trailblazers:

(Lines 66-87) The examination of a constellation of definitions, interpretations and constructions pertaining to the term learning style could provide a solid footing for the related studies, through which the understanding of it can be enriched and extended. Unlike learning strategies/methods that refer to the technique used in study, learning style is broadly defined as the inherent preferences of individuals as to how they engage in the learning process [2], and the “cognitive, affective and physiological traits” of students have received special attention [11]. To date, there has been a proliferation of learning style definitions proposed to explain people’s learning preferences, each focusing on different aspects. Efforts to dissect learning style have been contested, with some highlighting the dynamic process of the learner’s interaction with the learning environment [11] and others underlining the individualized ways of information processing [12]. One vivid explication involved the metaphor of an onion, pointing out the multilayer nature of learning styles. It was proposed that the outermost layer of the learning style could change in accordance with the external environment, while the inner layer is relatively stable [13-14]. In addition, a plethora of models germane to learning styles, including the experimental learning model [15], the multiple intelligences model [16] and the Felder-Silverman learning style model (FSLSM) [17], have provided contributive analytical lenses for related research. Despite the great abundance of the constructions of learning style models, the FSLSM, which consists of four paired dimensions, was utilized in this analysis due to its precise diagnosis of personal learning styles and its compatibility with a computer-assisted learning environment [18].

Considering the development of educational technology, the following paragraph provides the review of the research related to students’ learning styles in the web-based/blended learning environment:

(Lines 117-130) Eliminating the barrier of space and time, web-based technologies has become integrated in individuals’ daily lives and learning habits. Online and blended learning have begun to pervade virtually every aspect of the education landscape [30], and this warrants close attention. In addition to a series of studies that reflected upon the application of information and communication technology in the learning process [35-36], recent studies have found a mixed picture of whether students in a web-based/blended learning environment have a typical preference for learning. Based on the Visual, Aural, Reading or Write and Kinesthetic model, a comprehensive study scrutinized the learning styles of K12 students in a blended learning environment [37]. Researchers have investigated the learning styles of college students and concluded that online participation is a contributing factor to college students’ learning styles [38]. A recent study underscored the negative effects of kinesthetic learning style, whereas the positive effects of visual or auditory learning styles on students’ academic performance, were also marked in the context of blended learning [39].

The next paragraph delves into the studies centering on the effects of academic discipline in a blended learning environment, which represent the intersection of these two forces:

(Lines 131-149) Considering that academic disciplines and learning environment are both potent predictors of students’ learning styles, many studies have also concentrated on the effects of academic discipline in a blended learning environment. Focusing on college students’ learning styles in a computer-based learning environment, an inspiring study evaluated the different learning styles of web learners, namely, visual, sensing, global and sequential learners, in online courses. According to the analysis, compared with students from other colleges, liberal arts students, are more susceptible to the uneasiness that may result from remote teaching because of their learning styles [40]. A similar effort was made with the help of the CMS tool usage logs and course evaluations to explore the learning styles of disciplinary quadrants in the online learning environment. The results indicated that there were noticeable differences in tool preferences between students from different domains [41]. In comparison, within the context of blended learning, a comprehensive study employed chi-square statistics on the basis of the Community of Inquiry (CoI) presences framework, arguing that soft-applied discipline learners in the blended learning environment prefer the kinesthetic learning style, while no correlations between the learning style of soft-pure and hard-pure discipline students and the CoI presences were identified. However, it is noted that students’ blended learning experience depends heavily on academic discipline, especially for students in hard-pure disciplines [42].

We felt sorry that we failed to add review of the scholarly works about the learning styles of EFL students. This is because according to the suggestions of Reviewer 2, we have revised the title of our paper from “Differentiating the learning styles of EFL students with different disciplines in a blended learning setting: Insights from a machine learning-based algorithm” to “Differentiating the learning styles of college students with different disciplines in a blended learning setting: Insights from a machine learning-based algorithm”, where the word “EFL” was deleted and “College” was added accordingly. There are two reasons for this change. First, although our data were collected during the English language courses, students were actually from different disciplinary backgrounds, exhibiting learning styles which could be applicated in any subject. Second, some of the questions like “When I solve math problems …” in the Index of Learning Styles (ILS) questionnaire that we used in this research in fact also reflects students’ general learning styles rather than EFL learning styles. Accordingly, this modification has led to a series of revisions of the remaining part of the manuscript.

Even though, according to Reviewer 1’s suggestions, we still conducted the literature review to answer the suggestion “What are the major learning styles of EFL learning?”, which could broaden our understanding of the tailored nature of learning styles. The literature review was provided here in gray font because it was not included in the manuscript.

*Learning styles in EFL education*

In the context of EFL education, learning styles play crucial roles in clarifying students’ learning processes (e.g., Hsu, 2016), informing instructional strategies (e.g., Anna & Stracke, 2016), and predicting learning performance (e.g., Kuo, Chu & Huang, 2015). Although the acquisition of English language proficiency is the common goal pursued by EFL learners, learners differ in their own English language learning styles. Various studies have explored learning styles in relation to EFL learning, including students’ perceptual learning style preferences (e.g., Ma & Oxford, 2014; Shen, 2010), the frequent use of certain learning styles over other learning styles (e.g., Psaltou-Joycey & Kantaridou, 2011), the influence of learning styles on students’ strategy choice (e.g., Carson & Longhini, 2002; Ehrman, Leaver & Oxford, 2003; Kharb, Samanta, Jindal, & Singh, 2013), and the role of culture in shaping learning styles (e.g., Chuenjitwongsa, Bullock & Oliver, 2016; Ma & Oxford, 2014; Naserieh & Sarab, 2013; Wintergerst, DeCapua & Verna, 2003), just to name to a few.

For a specific look at the interconnections between EFL students’ learning styles and their disciplinary backgrounds, a positive relationship between visual and auditory learning styles has been found in Korean elementary and high school EFL students’ English language proficiency, whereas the kinesthetic learning style is recognized as a negative indicator (Kim & Kim, 2014), a finding that was previously confirmed by Bailey, Onwuegbuzie and Dailey (2000) with American college students who took French or Spanish as their second or foreign languages. Focusing on Taiwanese EFL learners’ styles during a lexical inference study across three disciplinary areas, namely, the English language, information management and mechanical engineering, Shen (2010) found that students with a group learning style often outperformed other students in terms of their word guessing power. The perceptual learning style preferences of Iranian EFL/ESL learners were kinaesthetic and tactile, with students in the technical fields showing a tactile tendency significantly more than those in the social sciences (Naserieh et al., 2013). The overall learning style preferences of Greek undergraduate EFL learners have been identified as “visual, extroverted, intuitive-random, closure-oriented and global” (Psaltou-Joycey & Kantaridou, 2011, p. 107), irrespective of other influencing factors. The literature can be seen as a testament to the recognition of the significant impact of learning styles on EFL learning and the differentiation of learning styles that vary in various contexts.

However, there has been a disproportionate lack of relevant literature that investigates EFL students’ learning style preferences, especially when the gigantic EFL population is considered (Evans & Zhang, 2013). Cheng and Guan (2015) conducted study on college students’ thinking and learning styles in various disciplines (educational technology, psychology, and elementary education) and found that EFL learners tended to be more holistic and structure-oriented in their thinking and learning. Huang, Hoi and Teo (2018) explored EFL undergraduates’ learning style preferences and found that the visual style was most preferred by the students. However, no relationship was shown between learning style preferences and their English language achievement. This finding stands in contrast to Sun and Teng’s (2017) finding of positive relationships between auditory and visual learning styles and students’ perceived language proficiency in their study on Chinese as a second language learners. Considering the importance of EFL education and the population of EFL learners, the extant studies present but a haphazard landscape of learning styles research, and few consistent and generalizable findings are currently available to illuminate future EFL education. Therefore, more studies in the EFL context are necessary.

### Comment 3b

(b) “How could disciplines influence learning styles?”

**Response to comment 3b**

Many thanks to the reviewer for this constructive suggestion. Following the definition of the key term “learning style”, literature in the **Introduction** section revolve around the influencing factors of learning styles, especially those with regard to academic disciplines:

(Lines 99-116) The past few decades have witnessed the emergence of abundant stimulating works focusing on various contextual factors that interact with learning styles. These factors include but are not limited to age [19], gender [20], personality [2, 21], learning environment [22] and learning experience [23]. Indeed, individuals’ learning styles are recognized as their adaptive orientation to learning and not fixed personality traits and can vary in diverse contexts accordingly; thus, related research in different contexts is vital to understanding learning styles in greater depth. In the higher education context, the academic discipline prominently represents an omnipresent variable that influences students’ distinctive learning styles, which echoes a multitude of investigations. A great many scholars have conducted meticulous investigations by focusing on the learning style of students of a single subject, such as engineering [24-25], medical science [26] and nursing science [27], to name but a few. Comparative studies between two/three/five/seven different disciplines could also be found, among which the preferences for learning of students majoring in different domains were carefully examined [28-32]. Taking students from multiple disciplines into consideration, a number of researchers have reflected on this topic from a more comprehensive perspective. One notable study explored the learning styles of students from 4 clusters of disciplines in an academic English language course, proposing that academic discipline is a significant predictor of students’ learning styles, with students from the soft-pure, soft-applied, hard-pure and hard-applied disciplines each favoring different modes of learning [33]. In particular, using the Inventory of Learning Styles (ILS) questionnaire, researchers also found prominent disparities in learning styles between students from four different disciplinary backgrounds in the special educational field of vocational training [34]. These works have found significant differences between the learning styles of students from different academic disciplines, thus giving support to the idea that learning style could be domain-dependent. Therefore, attention directed towards learning style research based on academic disciplines seems to be gaining increasing traction in recent years due to widespread recognition of the importance of individualized education.

### Comment 3c

(c) “What are the explicit limitations of SPSS which results in the incapability of it to address the present research problems?” A rigid theoretical framework would also be helpful in the analysis of the research results so as to make the discussion section better-structured and more in-depth.

**Response to comment 3c**

Our heartfelt thanks go to Reviewer 1 for the invaluable and constructive suggestions. Following the reviewer’s suggestions, we did carefully explain the reason why in this paper we selected SVM over SPSS ANOVA.

SPSS is a strong data analysis software through which a series of statistical analysis such as ANOVA could be performed. When it comes to the examination of the effect on a single variable, SPSS ANOVA could produce satisfactory results. However, SVM, a classic data mining algorithm, outperforms ANOVA in a dataset where a large number of variables with multidimensions intertwined and combined/collective effects of them were exerted on the classification results. In this study, the research objective was to ultimately detect an optimal set of key features collected from the overall 44 candidate variables that is needed to characterize different groups of students. Alone, a feature may not be significant enough to discriminate between students with different disciplinary background, but the effects of multiple key features may synergistically classify each two groups of students. Therefore, this study attempts to explore the collaborative effect of key contextual factors on the differentiation of students for different disciplinary background by examining a large number of contextual factors with the help of SVM machine learning algorithm.

A rationale of the classifier selection was explicitly made, which were also provided as below:

(Lines 165-207) Another issue addressed in this study is the selection of an optimal measurement that can effectively identify and differentiate individual learning styles [43]. The effective identification and differentiation of individual learning styles can not only help students develop greater awareness of their learning but also provide teachers with the necessary input to design tailor-made instructions in pedagogical practice. Currently, there are two general approaches to identify learning styles: a literature-based approach and a data-driven approach. The literature-based approach tends to borrow established rules from the existing literature, while the data-driven approach tends to construct statistical models using algorithms from fields such as machine learning, artificial intelligence, and data mining [44]. Research related to learning styles has been performed using predominantly traditional instruments, such as descriptive statistics, Spearman’s rank correlation, coefficient R [29], multivariate analysis of variance [40] and analysis of variance (ANOVA) [28, 34, 41-42]. Admittedly, these instruments have been applied and validated in numerous studies, in different disciplines, and across multiple timescales. Nevertheless, some of the studies using these statistical tools did not identify significant results [29, 45-46] or reached only loose conclusions [47]; this might be because of the inability of these methods to probe into the synergistic effects of variables. However, the limited functions of comparison, correlation, prediction, etc. are being complemented by a new generation of technological innovations that promise more varied approaches to addressing social and scientific issues. Machine learning is one such approach that has received much attention in both academia and beyond. As a subset of artificial intelligence, machine learning deals with algorithms and statistical models on computer systems, performing tasks based on patterns and inference instead of explicit instruction. As such, it can deal with high volumes of data at the same time, perform tasks automatically and independently, and continuously improve its performance based on past experience [46]. The use of similar machine learning approaches has also been proposed and tested by different scholars to identify students’ learning styles, with varying results regarding the classification of learning styles. For instance, a study examined the precision levels of four computational intelligence approaches: artificial neural network, genetic algorithm, ant colony system and particle swarm optimization, with a precision level of learning styles differentiation between 66% and 77% on average [48]. Another study was conducted to classify learning styles through SVM, the accuracy levels of which ranged from 53% to 84% [49]. These classic statistical approaches, despite showing some advantages compared to traditional statistical methods, generally shows that there is still great potential for further improvement of learning styles differentiation. In addition, the prediction performance comparison between SVM and artificial neural networks was conducted and found that SVM has a higher accuracy prediction than the latter [50]. This was further supported by another study, which yielded a similar result between SVM and the particle swarm optimization algorithm [51]. Moreover, when complemented by a genetic algorithm [52] and ant colony system [53], SVM has also shown improved results. These findings across different fields point to the reliability of SVM as an effective statistical tool for identification and differentiation analysis.

……

(Lines 349-365) Then, a traditional ANOVA was performed to examine whether there was a main effect of academic discipline on Chinese students’ learning styles. ANOVA could be performed using SPSS, a strong data analysis software that supports a series of statistical analyses. In regard to the examination of the effect of a single or few independent variables, SPSS ANOVA can produce satisfactory results. However, SVM, a classic data mining algorithm, outperforms ANOVA in a dataset where a large number of variables with multidimensions intertwined and their combined/collective effects are exerted on the classification results. In this study, the research objective was to efficiently differentiate and detect the key features among the 44 factors. Alone, a single factor or few factors might not be significant enough to discriminate the learning styles among the different disciplines. Selected by the SVM, the effects of multiple features may collectively enhance the classification performance. Therefore, the reason for selecting SVM over ANOVA is that in the latter case, the responses on all questions in a single dimension are summed instead of treated as individual scores; thus, the by-item variation is concealed. In addition, the SVM is especially suitable for statistical analysis with high-dimensional factors (usually > 10; 44-dimensional factors were included in this study) and can detect the effects collectively imposed by a feature set [73].

……

(Lines 208-226) Different students receive, process, and respond to information with different learning styles. A theoretical model of learning style can be used to categorize people according to their idiosyncratic learning styles. In this study, the FSLSM was adopted as a theoretical framework to address the collective impacts of students’ learning style differences across different disciplines (see Fig 1) because it is used very often in learning styles related to advanced learning technologies.

**Fig 1. The adapted Felder-Silverman learning style model.**

*Note.* This model specifies the four dimensions of the construct of learning style: visual/verbal, sensing/intuitive, active/reflective, and sequential/global. These four dimensions correspond to four psychological processes: input, perception, processing, and understanding.

The Felder-Silverman model includes learning styles scattered in four dimensions. Visual learners process information best when it is presented as graphs, pictures, etc., while verbal learners prefer spoken cues and remember best what they hear. Sensory learners like working with facts, data, and experimentation, while intuitions prefer abstract principles and theories. Active learners like to try things and learn through experimentation, while reflective learners prefer to think things through before taking action. Sequential learners absorb knowledge in a linear fashion and make progress step by step, while global learners tend to grasp the big picture before filling in all the details.

### Comment 4

2) It is suggested to point out the research questions clearly.

**Response to comment 4**

This important advice is highly appreciated. The Accordingly, we have added a section about the explanations of the two research objects and three research questions, which is presented in blue font as follows:

(Lines 227-240) In addition, by drawing from the above research, much of the existing literature on learning style models in the blended learning environment has focused on comparisons of student learning styles in one or some specific disciplines. There are few studies that adequately identifies the specific levels for differentiating students by discipline based on their learning styles. Therefore, a comprehensive investigation across the four general disciplines in Biglan’s taxonomy is still needed. Given the existence of the research gaps discussed above, this exploratory study seeks to address the following questions:

1) Can students’ learning styles be applied to differentiate various academic disciplines in the blended learning setting? If so, what are the levels of differentiability among different academic disciplines based on students’ learning styles?

2) What are the key features that can be selected to determine the collective impact on differentiation by a machine learning algorithm?

3) What are collective impacts of optimal feature sets?

### Comment 5

3) It is suggested to add a section about the practical implications for future researchers and educators.

**Response to comment 5**

We are extremely appreciative of the reviewer’s detailed and professional questions and suggestions. The practical implications are thus included in the last paragraph, and are listed below in blue font as below:

(Lines 719-732) In conclusion, the findings of this research should be considered not only a validation of previous findings but also an important reference in the field of blended learning. This study also serves as a resource for scholars who are dedicated to knowing more about the field of learning style and who may previously, have developed a thorough and in-depth understanding of this field that, perhaps, represents only the tip of the iceberg of possible knowledge. Furthermore, these findings may bridge the gap between research and reality, as they can help learners develop a more profound understanding of their blended learning tendencies, assist teachers in determining how to bring students’ learning styles into full play pedagogically in the context of blended learning, and support teachers in designing tailor-made instructions in the classrooms, which has been proven to be beneficial to students’ learning performance [117-119]. Furthermore, this study plays a positive role in promoting the realization of student-centered and personalized teaching, and students with different learning styles can also adapt to the current blended teaching environment to achieve satisfactory performance.

### Comment 6

The paper is well-presented in general, in spite of a few points in need of further improvement. For example, in the 1st paragraph, the statement “Knowing a student’s learning style has many obvious benefits” may not be in an intelligible fashion a s expected on a research paper. The authors are suggested to make revisions to the language.

**Response to comment 6**

Thanks to the reviewer for pointing out the imprecision in our language. After rewriting almost all of the introduction part, we have deleted the statement “Knowing a student’s learning style has many obvious benefits”. During the revision, we carefully proof read the entire manuscript and improved the language by asking our colleagues to review the main text and supporting materials. The native language of our colleagues is English, and they are all from my research fields in my university. Based on their domain-specific suggestions, we made necessary modifications and improvements to the manuscript, which are highlighted in blue font in the revised manuscript. We hope that the language of the manuscript now meets publication standards.

## Responses to Reviewer #2

### Comment 1

This manuscript tested the precision of different computational approaches in identifying and differentiating college sophomore students’ learning styles based on the Felder–Silverman model. Results showed that although no difference was found in participants’ learn style across different disciplinary groups, SVM-RFE machine learning algorithm showed that learning styles can differentiate various disciplines in EFL students’ blended learning processes as confirmed by the multiple performance indicators and further, some questions in the ILS questionnaire carry more substantive information about students’ learning styles than others. Overall, the manuscript is clearly written and the statistics are robust;

**Response to comment 1**

We would like to thank the reviewer for this positive comment.

### Comment 2

I have the following concerns regarding the theoretical assumption and data interpretation of the study before its acceptance for publication.

**Response to comment 2**

Many thanks to the reviewer for pointing out this problem. In the following parts, we will be presenting our point-by-point responses to the reviewers’ questions.

### Comment 3

A concern is that all the statistical comparisons of the current manuscript are based on the basic assumption that disciplinary differences predict learning style differences. Results showed that SVM-REF machine learning algorithm can be applied to show high percentage of correspondence between learning style and disciplinary backgrounds in some comparisons, such as HA-HP groups, whereas almost no such correspondence was found with ANOVA comparisons, which revealed no significant difference in learning styles across different participant groups. established on this, the manuscript argued that the SVM-REF is logically a better method because it is closer to reflect truth. However, the fallacy here is that the “assumed TRUTH” may not be “the truth”.

**Response to comment 3**

Many thanks for Reviewer 2 to point this problem out, and greatly appreciate the respectable reviewer’s opportunity, particularly the very constructive suggestions, to help us modify and improve our manuscript. We felt sorry that we did not explain the existing literature-based evidence and rationale for the selection of tools clearly in the previous manuscript. According to the suggestion, the manuscript was amended to clarify the reasons why academic discipline is vital to students’ learning styles, why ANOVA could sometimes not produce significant results, and why we selected SVM over ANOVA.

In the revised version, we have added a section of literature review on investigations of the links between academic disciplines and the learning style. A host of studies, using different statistical analysis, has verified this hypothesis. Besides, we have also found a few of studies which could not prove the significant between the academic discipline and the learning style. These analyses, using tools like descriptive statistics, Spearman’s rank correlation, coefficient R, ANOVA and MANOVA, often failed to give proof to the original hypothesis. This is mainly because these approaches could not decode the synergistic effects of a group of variables. It can be inferred that a single factor or few factors might not be significant enough to discriminate the learning styles among the different disciplines, and thus calls for an instrument that can explore the collective effects of factors. Thus, it helps explain the reason why we found different results when using ANOVA and using SVM in this research.

SVM, a strong machine learning algorithm, can help to present a satisfactory differentiability capability and SVM-based recursive feature elimination (RFE) can detect the collective effects of a group of variables. The advantages of SVM over ANOVA is that in the latter case, the responses on all questions in a single dimension are summed instead of treated as individual scores; thus, the by-item variation is concealed. In addition, the SVM is especially suitable for statistical analysis with high-dimensional factors (usually > 10; 44-dimensional factors were included in this study) and is able to detect the effects collectively imposed by a factor set. Since our research objective was to identify the key factors of the learning style that collectively indicating different disciplinary background, SVM was selected in this study to classify a large amount of data collected from the students of different disciplines in a comprehensive university with complex variable. Furthermore, SVM-RFE was applied in this study to discover whether there is an optimal factor set that can generate significant result in the relation of the learning style and academic disciplines.

During the revision, we added interpretations in the summary of literature review and in the section of “**Data Analysis Method**”. Again, we would like to thank the reviewer for offering this opportunity to compare these two methods in our revised paper more explicitly, and the corresponding modifications were highlighted in blue font in the revised manuscript and also provided here in blue font as below.

The following part that was newly added in the section of research gap reports the existing limitations of some of the previous research which through the utilization of SVM could be solved:

(Lines 150-207) Overall, the research seems to be gaining traction, and new perspectives are continually introduced. The recent literature on learning styles mostly focuses on the exploration of the disciplinary effects on the variation in learning styles, and some of these studies were conducted within the blended teaching environment. However, most of the studies focused only on several discrete disciplines or included only a small group of student samples [24-32]. Data in these studies were gathered through specialized courses such as academic English language [33] rather than the compulsory courses available to students from all disciplines. Despite the fact that certain investigations indeed boasted a large number of samples [38], the role of teaching was emphasized rather than students’ learning style. In addition, what is often overlooked is that a large number of variables related to learning styles could distinguish students from different academic disciplines in a blended learning environment, whereas a more comprehensive analysis that takes into consideration the effects of a great quantity of variables related to learning styles has remained absent. Therefore, one goal of the present study is to fill this gap and shed light on this topic.

Another issue addressed in this study is the selection of an optimal measurement that can effectively identify and differentiate individual learning styles [43]. The effective identification and differentiation of individual learning styles can not only help students develop greater awareness of their learning but also provide teachers with the necessary input to design tailor-made instructions in pedagogical practice. Currently, there are two general approaches to identify learning styles: a literature-based approach and a data-driven approach. The literature-based approach tends to borrow established rules from the existing literature, while the data-driven approach tends to construct statistical models using algorithms from fields such as machine learning, artificial intelligence, and data mining [44]. Research related to learning styles has been performed using predominantly traditional instruments, such as descriptive statistics, Spearman’s rank correlation, coefficient R [29], multivariate analysis of variance [40] and analysis of variance (ANOVA) [28, 34, 41-42]. Admittedly, these instruments have been applied and validated in numerous studies, in different disciplines, and across multiple timescales. Nevertheless, some of the studies using these statistical tools did not identify significant results [29, 45-46] or reached only loose conclusions [47]; this might be because of the inability of these methods to probe into the synergistic effects of variables. However, the limited functions of comparison, correlation, prediction, etc. are being complemented by a new generation of technological innovations that promise more varied approaches to addressing social and scientific issues. Machine learning is one such approach that has received much attention in both academia and beyond. As a subset of artificial intelligence, machine learning deals with algorithms and statistical models on computer systems, performing tasks based on patterns and inference instead of explicit instruction. As such, it can deal with high volumes of data at the same time, perform tasks automatically and independently, and continuously improve its performance based on past experience [46]. The use of similar machine learning approaches has also been proposed and tested by different scholars to identify students’ learning styles, with varying results regarding the classification of learning styles. For instance, a study examined the precision levels of four computational intelligence approaches: artificial neural network, genetic algorithm, ant colony system and particle swarm optimization, with a precision level of learning styles differentiation between 66% and 77% on average [48]. Another study was conducted to classify learning styles through SVM, the accuracy levels of which ranged from 53% to 84% [49]. These classic statistical approaches, despite showing some advantages compared to traditional statistical methods, generally shows that there is still great potential for further improvement of learning styles differentiation. In addition, the prediction performance comparison between SVM and artificial neural networks was conducted and found that SVM has a higher accuracy prediction than the latter [50]. This was further supported by another study, which yielded a similar result between SVM and the particle swarm optimization algorithm [51]. Moreover, when complemented by a genetic algorithm [52] and ant colony system [53], SVM has also shown improved results. These findings across different fields point to the reliability of SVM as an effective statistical tool for identification and differentiation analysis.

In the section of “Data Analysis Method”, the practical advantages of using SVM over ANOVA in this specific study are further illustrated:

(Lines 349-365) Then, a traditional ANOVA was performed to examine whether there was a main effect of academic discipline on Chinese students’ learning styles. ANOVA could be performed using SPSS, a strong data analysis software that supports a series of statistical analyses. In regard to the examination of the effect of a single or few independent variables, SPSS ANOVA can produce satisfactory results. However, SVM, a classic data mining algorithm, outperforms ANOVA in a dataset where a large number of variables with multidimensions intertwined and their combined/collective effects are exerted on the classification results. In this study, the research objective was to efficiently differentiate and detect the key features among the 44 factors. Alone, a single factor or few factors might not be significant enough to discriminate the learning styles among the different disciplines. Selected by the SVM, the effects of multiple features may collectively enhance the classification performance. Therefore, the reason for selecting SVM over ANOVA is that in the latter case, the responses on all questions in a single dimension are summed instead of treated as individual scores; thus, the by-item variation is concealed. In addition, the SVM is especially suitable for statistical analysis with high-dimensional factors (usually > 10; 44-dimensional factors were included in this study) and can detect the effects collectively imposed by a feature set [73].

### Comment 4

No evidence showed such a correspondence between disciplinary background and learning style of ALL individuals. Learning styles have been shown to be related to a whole multitude of factors, such as individuals’ personality, leaning background, experience, etc. Disciplinary background is only one of them, maybe a minor one out of many. Thus, the “non-significant result” might be true here. While the “significant result” might not be the truth. Hidden in the sophisticated descriptions of all statistical methods and algorithms, the manuscript did not provide literature to support such a clear route and corresponding relationship between disciplinary background and learning style. Ref 24 is related but not directly supporting it.

**Response to comment 4**

We would like to thank the reviewer for pointing out the lack of literature from this aspect, we do agree that learning styles can interact with various contextual factors, such as individuals' personality, leaning background, experience, etc. During the revision, we provided a detailed section of literature review, which dealt with four related aspects, namely, the definition of learning style, the effects of academic discipline on learning styles, the effects of web-based/blended learning environment on learning styles, and the effects of academic discipline on learning styles in a web-based/blended learning environment, respectively. In a nutshell, we felt sorry that we did not provide sufficient literature to support our assumption, and the corresponding modifications were highlighted in blue font in the revised manuscript and the whole picture of the literature review is provided below in blue font for the ease of reference:

(Lines 55-149) Learning style, as an integral and vital part of students’ learning process, has always been a long-lasting and hot-debated topic in the field of education and pedagogy. Originally developed from the field of psychology, psychological classification, and cognitive research several decades ago [1], the term “learning style” is generally defined as the learner’s innate and individualized preference for ways of participation in learning practice [2]. Theoretically, learning style provides a window into students’ learning processes [3-4], predicts students’ learning outcomes [5-6], and plays a critical role in designing individualized instruction [7]. Knowing a student’s learning style and personalizing instruction to students’ learning style could enhance their satisfaction [8], improve their academic performance [9], and even reduce the time necessary to learn [10].

The examination of a constellation of definitions, interpretations and constructions pertaining to the term learning style could provide a solid footing for the related studies, through which the understanding of it can be enriched and extended. Unlike learning strategies/methods that refer to the technique used in study, learning style is broadly defined as the inherent preferences of individuals as to how they engage in the learning process [2], and the “cognitive, affective and physiological traits” of students have received special attention [11]. To date, there has been a proliferation of learning style definitions proposed to explain people’s learning preferences, each focusing on different aspects. Efforts to dissect learning style have been contested, with some highlighting the dynamic process of the learner’s interaction with the learning environment [11] and others underlining the individualized ways of information processing [12]. One vivid explication involved the metaphor of an onion, pointing out the multilayer nature of learning styles. It was proposed that the outermost layer of the learning style could change in accordance with the external environment, while the inner layer is relatively stable [13-14]. In addition, a plethora of models germane to learning styles, including the experimental learning model [15], the multiple intelligences model [16] and the Felder-Silverman learning style model (FSLSM) [17], have provided contributive analytical lenses for related research. Despite the great abundance of the constructions of learning style models, the FSLSM, which consists of four paired dimensions, was utilized in this analysis due to its precise diagnosis of personal learning styles and its compatibility with a computer-assisted learning environment [18].

The past few decades have witnessed the emergence of abundant stimulating works focusing on various contextual factors that interact with learning styles. These factors include but are not limited to age [19], gender [20], personality [2, 21], learning environment [22] and learning experience [23]. Indeed, individuals’ learning styles are recognized as their adaptive orientation to learning and not fixed personality traits and can vary in diverse contexts accordingly; thus, related research in different contexts is vital to understanding learning styles in greater depth. In the higher education context, the academic discipline prominently represents an omnipresent variable that influences students’ distinctive learning styles, which echoes a multitude of investigations. A great many scholars have conducted meticulous investigations by focusing on the learning style of students of a single subject, such as engineering [24-25], medical science [26] and nursing science [27], to name but a few. Comparative studies between two/three/five/seven different disciplines could also be found, among which the preferences for learning of students majoring in different domains were carefully examined [28-32]. Taking students from multiple disciplines into consideration, a number of researchers have reflected on this topic from a more comprehensive perspective. One notable study explored the learning styles of students from 4 clusters of disciplines in an academic English language course, proposing that academic discipline is a significant predictor of students’ learning styles, with students from the soft-pure, soft-applied, hard-pure and hard-applied disciplines each favoring different modes of learning [33]. In particular, using the Inventory of Learning Styles (ILS) questionnaire, researchers also found prominent disparities in learning styles between students from four different disciplinary backgrounds in the special educational field of vocational training [34]. These works have found significant differences between the learning styles of students from different academic disciplines, thus giving support to the idea that learning style could be domain-dependent. Therefore, attention directed towards learning style research based on academic disciplines seems to be gaining increasing traction in recent years due to widespread recognition of the importance of individualized education.

Eliminating the barrier of space and time, web-based technologies has become integrated in individuals’ daily lives and learning habits. Online and blended learning have begun to pervade virtually every aspect of the education landscape [30], and this warrants close attention. In addition to a series of studies that reflected upon the application of information and communication technology in the learning process [35-36], recent studies have found a mixed picture of whether students in a web-based/blended learning environment have a typical preference for learning. Based on the Visual, Aural, Reading or Write and Kinesthetic model, a comprehensive study scrutinized the learning styles of K12 students in a blended learning environment [37]. Researchers have investigated the learning styles of college students and concluded that online participation is a contributing factor to college students’ learning styles [38]. A recent study underscored the negative effects of kinesthetic learning style, whereas the positive effects of visual or auditory learning styles on students’ academic performance, were also marked in the context of blended learning [39].

Considering that academic disciplines and learning environment are both potent predictors of students’ learning styles, many studies have also concentrated on the effects of academic discipline in a blended learning environment. Focusing on college students’ learning styles in a computer-based learning environment, an inspiring study evaluated the different learning styles of web learners, namely, visual, sensing, global and sequential learners, in online courses. According to the analysis, compared with students from other colleges, liberal arts students, are more susceptible to the uneasiness that may result from remote teaching because of their learning styles [40]. A similar effort was made with the help of the CMS tool usage logs and course evaluations to explore the learning styles of disciplinary quadrants in the online learning environment. The results indicated that there were noticeable differences in tool preferences between students from different domains [41]. In comparison, within the context of blended learning, a comprehensive study employed chi-square statistics on the basis of the Community of Inquiry (CoI) presences framework, arguing that soft-applied discipline learners in the blended learning environment prefer the kinesthetic learning style, while no correlations between the learning style of soft-pure and hard-pure discipline students and the CoI presences were identified. However, it is noted that students’ blended learning experience depends heavily on academic discipline, especially for students in hard-pure disciplines [42].

### Comment 5

Without consideration of all other factors and only entering the single disciplinary factor into the algorithm arbitrarily is not substantiated.

**Response to comment 5**

Thanks to the reviewer for pointing out this important problem. According to Walberg, the influencing factors of people’s learning are varied. Just as the reviewers pointed out that learning styles have been shown to be related to a whole multitude of factors, such as individuals’ personality, leaning background, experience, etc., we pretty agree with this idea and found many studies in reference to these factors, and listed them in the literature review. Given the fact that there are a multitude number of researches on these directions, while limited attention has been paid to the impact of different academic disciplines on learning styles, the current research attempt to fill in this research niche by focusing on the variable of disciplines. We do appreciate reviewer’s valuable suggestions of considering other factors and the joint effect of other contextual factors with academic disciplinary background on learning styles, but what frustrated us was due to the lack of relevant data at present, the discussion of these factors was not unfolded. Thanks for the reviewer’s inspiration, we are very interested in and working on delving into these directions in our future study. In the next step, we set about taking other personal and contextual factors into consideration to make our analysis more comprehensive. Based on the valuable comments, we sincerely point the problem out in the **Limitation** session, and also provide some suggestions for future researches in the last paragraph of the manuscript, which were highlighted in blue font in the manuscript and were also provided here as below:

(Lines 708-718) Despite the notable findings of this study, it is subject to some limitations that may be perfected in further research. First, the current analysis examined the learning styles without allowing for the effects of other personal or contextual factors. The educational productivity model proposed by Walberg underlines the significance of the collected influence of contextual factors on individuals’ learning [115]. For example, teachers from different backgrounds and academic disciplines are inclined to select various teaching methods and to create divergent learning environments [116], which should also be investigated thoroughly. The next step is therefore to take into account the effects of educational background, experience, personality and learning experience to gain a more comprehensive understanding of students’ learning process in the blended setting.

### Comment 6

The results did show non-significant (relatively low performance) in some backgrounds. In the discussion the authors attributed this to the fact that these students chose courses from other disciplines and that lecturing in EFL classrooms regardless of the students’ background information diminished their disciplinary differences (Line 388-390). Since all students are in such a protocol, I am thinking why there are significant results in other discipline comparisons.

**Response to comment 6**

Thanks to the reviewer for pointing out the imprecision in our arguments. During our revision, we did carefully reconsider the reason why the result showed non-significant (relatively low performance) between the SA-SP differentiation. After careful consideration, it come to the conclusion that the initial reason that students with different disciplinary background taking the same EFL classes may diminish their disciplinary differences is not strong enough to account for the result, because the compulsory classes such as English courses only occupies a little proportion of the whole courses that students take, so the limited effect might not account for the finding. In light of this, we agree that this layer of analysis can be deleted, and after reviewed the literature of the related question comprehensively, we were inspired to come up with a new layer of analysis which traces back to these student’s high school learning experience. As far as Chinese high school students are concerned, majority of them were required to choose either liberal art (composed of history, politics and geography disciplines) or natural science (composed of mathematics, physics and chemistry disciplines) as their learning subjects apart from the compulsory subjects, namely Chinese language, Mathematics, and English language. This choice directly restrict the disciplines that they can choose in the higher education, to be specific, according to Biglan’s classification scheme of academic disciplines, soft applied (e.g. Economics, Education) and soft pure (e.g. Psychology, Sociology) disciplines mostly consist of students who chose social science in high school, whereas, hard applied (e.g. Engineering, Computer Science) and hard pure (e.g. Chemistry, Physics) disciplines is mostly composed of students whose choices were on natural science in high school. In regarding to this background, it is facile to conclude that no matter soft or hard disciplines, the students of which all shared two kinds of similar discipline backgrounds during the high school studies, which might explain the sharp contrast between soft and hard disciplines, but comparatively modest one between pure and applied disciplines. Furthermore, previous research also proved that the difference of pure and applied disciplines within the soft disciplinary domain is significantly smaller than that within the hard one, for example, regarding to the term of “I learn more when studying in a group”, the effect size (r^2^) between soft pure and soft applied disciplines is 0.04 indicating a small difference, while that between hard pure and hard applied disciplines is 0.09 indicating a medium difference. The above findings underpin the current finding in this manuscript, explaining why while other three models (i.e. HP-HA, HP-SA, SP-HA) generate significant results, the result of SP-SA model is not significantly different. The modifications were provided below with blue font:

(Lines 566-590) Regarding low differentiability, one explanation might be the indistinct classification of some emerging “soft disciplines”. It was noted that psychology, for example, could be identified as “a discipline that can be considered predominantly ‘soft’ and slightly ‘purer’ than ‘applied’ in nature” [98] (p. 43-53), which could have blurred the line between the SA and SP disciplines. As there is now no impassable gulf separating the SA and SP disciplines, their disciplinary differences may have diminished in the common practice of lecturing in classrooms. Another reason comes from the different cultivation models of “soft disciplines” and “hard disciplines” for sample students. In their high school, sample students are generally divided into liberal art students and science students and are then trained in different environments of knowledge impartation. The two-year unrelenting and intensive training makes it possible for liberal art students to develop similar thinking and cognitive pattern that are persistent. After the college entrance examination, most liberal art students select SA or SP majors. However, a year or more of study in university does not exert strong effects on their learning styles, which explains why a multitude of researchers have traditionally investigated the SA and SP disciplines together, calling them simply “social science” or “soft disciplines” compared with “natural science” or “hard disciplines”. There have been numerous contributions pointing out similarities in the learning styles of students from “soft disciplines” [30, 99-100]. However, students majoring in natural science exhibit considerable differences in learning styles, demonstrating that the talent cultivation model of “hard disciplines” in universities is to some extent more influential on students’ learning styles than that of the “soft disciplines”. However, further compelling interpretations of this phenomenon await only the development of a sufficient level of knowledge accumulation among scholars in this area.

### Comment 7

My other concern is that although the title claims “learning styles of EFL students” explicitly, the current data is not in essence related specifically to blended EFL learning. The questionnaire adopted in the current study is tapping into learners’ general learning style. The only thing that is related to EFL is that questionnaires were collected in EFL class time period. Such items as “When I solve math problems …” (P12) in the questionnaire show that is not specifically EFL learning style.

**Response to comment 7**

Thank you very much for pointing out the confusion of having EFL as the modifier of students. In the previous submission, considering the samples collected were among the EFL students who taking blended learning for English courses compulsorily, we add this as a background information, but what we intended to investigate was the relation between the features of learning styles and different academic disciplines which is not essentially related to the language background of sample students.

The reviewer’s helpful suggestions have drawn us out of the mind box of fallacies. First, although our data were collected during the English language courses, students were actually from different disciplinary backgrounds, exhibiting learning styles which could be applicated in any subject. Second, some of the questions like “When I solve math problems …” in the Index of Learning Styles (ILS) questionnaire we used in this research in fact also reflects students’ general learning styles rather than EFL learning styles. We have thus revised the title of our paper from “Differentiating the learning styles of EFL students with different disciplines in a blended learning setting: Insights from a machine learning-based algorithm” to “Differentiating the learning styles of college students with different disciplines in a blended learning setting: Insights from a machine learning-based algorithm”, where the word “EFL” was deleted and another word “College” was added. After this adjustment, the manuscript can not only better elaborate our initial thought on revealing the relation between learning styles and academic disciplines with students’ academic disciplinary background covering a wide spectrum of a comprehensive university, but also be more constructive for related researched on sample students with other learning background. Thanks again for this valuable advice rendering our manuscript more explicit and logical. Accordingly, this modification has led to a series of revisions of the discussion in the remaining part of the manuscript.

### Comment 8

Line 63-65: Ref 26 is emphasizing the importance of real-world models instead of using machine learning blindly. However, this paragraph here the authors are highlighting the merits of machine learning. the logic is not consistent here.

**Response to comment 8**

We would like to thank the reviewer for pointing out this fault very much. After re-examine the Ref 26 in the original manuscript, we found it irrelevant to the main idea of this paragraph, that is, highlighting the merits of machine learning. During our revision, this inappropriate reference was deleted, so that the prior sentence and the subsequent sentence could be more logically connected.

### Comment 9

Table titles can be shortened. For example, in table 2 such words as “Constructed in This Study” are redundant.

**Response to comment 9**

The reviewer’s comment is greatly appreciated. We felt sorry that we did not make our language concise here. According to this suggestion, we have shortened the title of Table 2 from “Classification Capacity of the Different Models Constructed in This Study as Measured by Five Performance Indicators” to “Classification Capacity of the Different Models”. Accordingly, the title of Table 3 has also been chopped from “Classification Capacity of Different Models Constructed based on 20 Selected Features as Measured by the Five Performance Indicators” to “Classification Capacity of Different Models Constructed based on 20 Selected Features”, to avoid redundancy.
