# Supplementary material for: Differentiating the learning styles of college students in different disciplines in a college English blended learning setting
Source: PLoS One. 2021 May 20;16(5):e0251545. doi: 10.1371/journal.pone.0251545 (PMC8139239; doi:10.1371/journal.pone.0251545)
Supplement: Attachment — Submitted filename: Authors Responses to Reviewers Comments.docx [file pone.0251545.s005.docx]

**Authors’ Responses to Reviewers’ Comments**

To,

The Editor and the Esteemed Reviewers,

PLOS ONE,

Sub: Submission of revised manuscript (Manuscript ID: PONE-D-20-14494R1)

Dear Professor Xie and Esteemed Reviewers,

We would like to express our sincere gratitude for reviewing our manuscript entitled “Differentiating the learning styles of college students with different disciplines in a college English blended learning setting” and providing us an opportunity to make corrections in it. We have revised our manuscript according to the valuable comments of you and the reviewers. The following is an outline of our point-to-point responses and corresponding revisions made to our manuscript.

Once again, we are thankful to all of the reviewers for promote the quality of our manuscript!

Best regards & Happy new year,

Authors

**Responses to Reviewers’ Comments**

Table of Contents

[Responses to Reviewer #1	1](#_Toc60071897)

[Comment 1	1](#_Toc60071898)

[Comment 2	1](#_Toc60071899)

[Comment 3	1](#_Toc60071900)

[Comment 4	4](#_Toc60071901)

[Comment 5	5](#_Toc60071902)

[Comment 6	5](#_Toc60071903)

[Comment 7	6](#_Toc60071904)

[Comment 8	7](#_Toc60071905)

[Responses to Reviewer #2	9](#_Toc60071906)

[Comment 1	9](#_Toc60071907)

[Comment 2	9](#_Toc60071908)

**Responses to Reviewer #1**

**Comment 1**

The authors have carefully revised this paper and addressed all the comments. Remarkable improvements are spotted.

**Response to comment 1**

Many thanks to the reviewer for this positive comment.

**Comment 2**

The title is very informative but can be shorter and catchier.

**Response to comment 2**

Thanks to Reviewer 1 for this recommendation. We have shortened the title “Differentiating the learning styles of college students with different disciplines in a blended learning setting: Insights from a machine learning-based algorithm” to “Differentiating the learning styles of college students with different disciplines in a college English blended learning setting” to make it precise while still informative enough.

**Comment 3**

The introduction is a little bit mixed with the literature review. Since this paper involves several different topics, I suggest the authors divide the current “Introduction” into several sections so your readers can follow you more easily. I suggest a division as follows: (1) a short section for a catchy introduction briefly specifying the research background, research rationales and research questions; (2) a section for literature review, in which the descriptions of the theories on learning styles and blended learning are organized separately in different sub-sections and catchy sub-titles; (3) a section for research gaps and research questions.

**Response to comment 3**

Many thanks to the reviewer for this invaluable suggestion. Since the format of PLoS ONE suggests only five Level 1 headings, namely, introduction, materials and methods, results, discussions, and conclusions; thus, we took the effort to divide the Level 1 introduction section into three Level 2 sub-sections, namely, research background, literature review and research gaps and research questions according to the suggestions to make it clearly structured and friendly readable.

(1) In the research background sub-section, we have briefly introduced the research rationale and research significance. Modifications were made in the manuscript as a newly developed section in blue font, which is also provided here as follows for the ease of reference:

**Research background**

Learning style, as an integral and vital part of students’ learning process, has always been a long-lasting and hot-debated topic in the field of education and pedagogy……

However, knowledge of the learning styles of students from different disciplines in blended learning environments is limited [11-13]. In an effort to address this gap, the present study is designed to achieve two major objectives. First, it intends to gain insight on how disciplinary background impacts students’ learning styles in a blended learning environment based on data collected in a compulsory college English course in which students across 46 disciplines were enrolled. Second, it introduces a novel machine learning method named the support vector machine (SVM) to the field of education to identify an optimal set of factors that can simultaneously differentiate students of different academic disciplines. This research aims to provide a clearer picture of the relationship between disciplinary factors and the identification of students’ learning styles in a blended learning setting.

(2) In the literature review sub-section, we have divided the original mixed and confusing part into two sub-sections with clear sub-titles, where the theories of learning style and blended learning were examined successively. In the first Level 3 sub-section entitled “Theories of learning styles”, definitions, influencing factors and models of learning styles were investigated. The examination of the conceptual framework of the research, namely, Felder-Silverman learning style model (FSLSM) was also included. In the second Level 3 sub-section named “Learning styles in an online/blended learning environment”, scholarly works with regard to learning styles in both web-based environment and blended learning environment were examined. The revised part is marked in blue font and is presented here as follows:

**Literature Review**

**Theories of learning styles**

The examination of a constellation of definitions, interpretations and constructions pertaining to the term learning style could provide a solid footing for the related studies, through which the understanding of it can be enriched and extended……

Learning styles vary according to a series of factors, including but not limited to age [22], gender [23], personality [2, 24], learning environment [25] and learning experience [26]. In the higher education context, the academic discipline prominently represents an omnipresent variable that influences students’ distinctive learning styles, which echoes a multitude of investigations [27-35]. One notable study explored the learning styles of students from 4 clusters of disciplines in an academic English language course, ……

Different students receive, process, and respond to information with different learning styles. A theoretical model of learning style can be used to categorize people according to their idiosyncratic learning styles.…….

The FSLSM includes learning styles scattered among four dimensions. Visual learners process information best when it is presented as graphs, pictures, etc.,…….

**Learning styles in an online/blended learning environment**

Individuals’ learning styles reflect their adaptive orientation to learning and are not fixed personality traits. Consequently, learning styles can vary among diverse contexts, and related research in different contexts is vital to understanding learning styles in greater depth. Web-based technologies eliminate barriers of space and time and have become integrated in individuals’ daily lives and learning habits.…...

Considering that academic disciplines and learning environment are both potent predictors of students’ learning styles, many studies have also concentrated on the effects of academic discipline in a blended learning environment.……

(3) In the sub-section for research gaps and research questions, we have briefly summarized the research gaps of the abovementioned scholarly works and elaborated on the two main objectives. This section is now listed below for the reviewer’s reference, and the modifications were highlighted in blue font here and in the revised manuscript:

**Research gaps and research questions**

Overall, the research seems to be gaining traction, and new perspectives are continually introduced ……. Therefore, one goal of the present study is to fill this gap and shed light on this topic.

Another issue addressed in this study is the selection of an optimal measurement that can effectively identify and differentiate individual learning styles [43]……These findings across different fields point to the reliability of SVM as an effective statistical tool for identification and differentiation analysis.

Therefore, a comprehensive investigation across the four general disciplines in Biglan’s taxonomy using a strong machine learning approach is needed. Given the existence of the research gaps discussed above, this exploratory study seeks to address the following questions:

1) Can students’ learning styles be applied to differentiate various academic disciplines in the blended learning setting? If so, what are the levels of differentiability among different academic disciplines based on students’ learning styles?

2) What are the key features that can be selected to determine the collective impact on differentiation by a machine learning algorithm?

3) What are collective impacts of optimal feature sets?

**Comment 4**

The methodology is well written, with clear organization and sufficient justification.

**Response to comment 4**

We would like to thank the reviewer very much for this positive comment.

**Comment 5**

The authors may double check the use of punctuation. For example, in line 564, “soft discipline”. ️→ “soft discipline.”

**Response to comment 5**

Thanks to the reviewer for pointing out this problem. Indeed, according to the rules of punctuation, we should put the full stop in front of the quotation marks in Line 570. At the same time, similar problems were checked throughout the manuscript and were revised if necessary.

**Comment 6**

The coherence and logics within the paragraphs may be further improved, especially in the discussion and implication sections. For example, in line 581-587‬, the authors used “However” in two successive sentences, causing logical incoherence.‬‬‬‬‬‬‬‬‬‬‬‬

**Response to comment 6**

Many thanks to the reviewer for this constructive suggestion. We felt very sorry that we did not pay attention to the repeated use of the word “However” in the arguments in the original manuscript, which caused unnecessary misunderstandings. The first “However” in Line 587 was initially conceived to indicate a huge difference between two group of students, namely, students majoring in natural science and their liberal art counterparts. The second use of “However” was indicative of the insufficient knowledge about the causal effects of this interpretation. According to the suggestions, we have made revisions to this argument and tried to make it more applicable. We have kept the first “However” and changed the expression in Line 591. The revised sentence is provided below in blue font for reference:

However, students majoring in natural science exhibit considerable differences in learning styles, demonstrating that the talent cultivation model of “hard disciplines” in universities is to some extent more influential on students’ learning styles than that of the “soft disciplines”. Further compelling interpretations of this phenomenon await only the development of a sufficient level of accumulated knowledge among scholars in this area.

**Comment 7**

The authors raised many examples in the discussions, which is good. I suggest the authors put more specific explanations for their arguments in the discussions. Examples may be used as evidence to SUPPORT the arguments and explanations, rather than REPLACE them.

**Response to comment 7**

Our heartfelt thanks go to the reviewer for this invaluable and helpful suggestion. Following the reviewer’s suggestion, we did carefully add more specific explanations of the results, using examples as evidence to support our arguments. The revised part is provided below in blue font for ease of reference:

In addition, Questions 2, 4, 7, and 9 also occur frequently in the 20 features selected for the different models.…… Within this context, the significance of this question could also be explained by the psychological evaluation of “loss and gain”, as students’ different learning styles are associated with expected reward values and their internal motivational drives, which are determined by their personality traits [112]. When faced with the risk of “loosing face”, whether students will express their ideas in front of a group of people depends largely on their risk and stress management capabilities and the presence of an appropriate motivation system.

The other two questions also convey similar messages regarding personality differences.…... This might also be a vivid reflection of the multi-faceted effects of blended learning, which may differ in their consonance with the features of each discipline. First, teachers from different domains use technology in different ways, and student from different disciplines may view blended learning differently. For instance, the characteristics of soft-applied fields entail specialized customization in blended courses, further broadening the gulf between different subjects [116]. Second, although blended learning is generally recognized as a stimulus to students’ innovation [117], some students who are used to an instructivist approach in which the educator acts as a ‘sage on the stage’ will find it difficult to adapt to a social constructivist approach in which the educator serves as a ‘guide on the side’ [118]. This difficulty might not only negatively affect students’ academic performance but also latently magnify the effects of different academic disciplines.

**Comment 8**

Is the manuscript presented in an intelligible fashion and written in standard English?

PLOS ONE does not copyedit accepted manuscripts, so the language in submitted articles must be clear, correct, and unambiguous. Any typographical or grammatical errors should be corrected at revision, so please note any specific errors here.

Reviewer #1: No

**Response to comment 8**

Thank you very much for the reviewer’s valuable suggestion. During the revision, we carefully proof-read the entire manuscript and improved the language by asking our colleagues to review the main text and the supporting materials. The native language of our colleagues is English, and they are all from my research fields in my university. Base on their suggestions, necessary modifications and improvements to the manuscript were made, which were all highlighted in blue font in the revised manuscript.

In addition, this version of manuscript was sent to the professional proof-reading agency *American Journal Experts* (AJE) for English language editing, which is the official English language editing agency partner of PLOS. The proof-reading quality certificate (AJE Order NO.: MCF1YPS3) with the title of this manuscript was offered and also present here as below:

## Responses to Reviewer #2

### Comment 1

The revised manuscript has addressed all my concerns. The revised manuscript is improved significantly and should be a nice contribution to the literature.

**Response to comment 1**

Many thanks to the reviewer for this positive comment.

### Comment 2

The abstract is a big heavy in sentence structure, the authors may consider condense it a little bit before publication.

**Response to comment 2**

The reviewer’s valuable suggestion is greatly appreciated. According to the suggestion, we have condensed the abstract according to the suggestion and made the sentence structure more precise. The revised abstract is provided as below in blue font for the reviewer’s reference:

Learning styles are critical to educational psychology, especially when investigating various contextual factors that interact with individual learning styles. Drawing upon Biglan’s taxonomy of academic tribes, this study systematically analyzed the learning styles of 790 sophomores in a blended learning course with 46 specializations using a novel machine learning algorithm called the support vector machine (SVM). Moreover, an SVM-based recursive feature elimination (SVM-RFE) technique was integrated to identify the differential features among distinct disciplines. The findings of this study shed light on the optimal feature sets that collectively impacted the students’ learning style differences in a college English blended learning setting.
