# Supplementary material for: Differentiating the learning styles of college students in different disciplines in a college English blended learning setting
Source: PLoS One. 2021 May 20;16(5):e0251545. doi: 10.1371/journal.pone.0251545 (PMC8139239; doi:10.1371/journal.pone.0251545)
Supplement: Attachment — Submitted filename: Authors Responses to Reviewers Comments.docx [file pone.0251545.s006.docx]

**Authors’ Responses to Reviewer 1’s Comments**

To,

The Esteemed Editor and Reviewer 1,

PLOS ONE,

Sub: Submission of revised manuscript (Manuscript ID: PONE-D-20-14494R1)

Dear Professor Xie and Esteemed Reviewer 1,

We would like to express our sincere gratitude for reviewing our manuscript entitled “Differentiating the learning styles of college students in different disciplines in a college English blended learning setting” and providing us an opportunity to make corrections in it. We have revised our manuscript according to the valuable comments of the editor and the reviewer. The following is an outline of our point-to-point responses and corresponding revisions made to our manuscript.

Once again, we are thankful to all of the editor and reviewers for promoting the quality of our manuscript!

Best regards,

Authors

**Responses to Reviewer 1’s Comments**

**Table of Contents**

[Responses to Reviewer #1	1](#_Toc67036073)

[Comment 1	1](#_Toc67036074)

[Comment 2	1](#_Toc67036075)

[Comment 3	1](#_Toc67036076)

[Comment 4	2](#_Toc67036077)

[Comment 5	3](#_Toc67036078)

[Comment 6	4](#_Toc67036079)

[Comment 7	4](#_Toc67036080)

[Comment 8	5](#_Toc67036081)

[Comment 9	5](#_Toc67036082)

[Comment 10	6](#_Toc67036083)

[Comment 11	6](#_Toc67036084)

[Comment 12	7](#_Toc67036085)

[Comment 13	7](#_Toc67036086)

[Comment 14	9](#_Toc67036087)

[Comment 15	11](#_Toc67036088)

[Comment 16	12](#_Toc67036089)

[Comment 17	12](#_Toc67036090)

[Comment 18	15](#_Toc67036091)

[Comment 19	15](#_Toc67036092)

[Comment 20	17](#_Toc67036093)

[Comment 21	18](#_Toc67036094)

[Comment 22	18](#_Toc67036095)

[Comment 23	19](#_Toc67036096)

[Comment 24	20](#_Toc67036097)

**Responses to Reviewer #1**

**Comment 1**

Overall, this article was well written and organized. Additionally, great revisions have been made based on the suggestions by previous reviewers, which was made this article more convincing.

**Response to comment 1**

Many thanks to the reviewer for the generous remarks and the recognition of our work.

**Comment 2**

However, some details should be offered, and minor revisions should be made before publication. They are listed as follows:

**Response to comment 2**

Thanks for this valuable suggestion. In the following parts, we will be presenting our point-by-point responses to Reviewer 1’s comments.

**Comment 3**

1. Though this article has been proofread, some language concerns existed. The authors should carefully revise the language before re-submit the manuscript.

**Response to comment 3**

Many thanks to the reviewer for pointing out the language imprecision in the manuscript. During the revision, we carefully proof read the entire manuscript and improved the language by asking our colleagues to review the main text and supporting materials. The native language of our colleagues is English, and they are all from my research fields in my university. Based on their domain-specific suggestions, we made necessary modifications and improvements to the manuscript, which were highlighted in blue font in the revised manuscript. In addition, this version of manuscript was also sent to the professional proof-reading agency American Journal Experts (AJE) for English language editing, which is the official English language editing agency partner of PLOS. We hope that the language of the manuscript now meets publication standards.

**Comment 4**

(1) Inaccurate wording such as ‘impacted the students’ learning style differences (Abstract)’, ‘a clearer picture. (Line 66, what did the author compared with?)’, and ‘an omnipresent’.

**Response to comment 4**

The reviewer’s valuable suggestion is greatly appreciated. We feel sorry that the inaccurate wording in the original submission has perplexed the readers and caused unnecessary misunderstandings. During the revision, we have made modifications to these misuses of words according to the valuable suggestion:

1) The word “differences” has been removed from the original awkward expression ‘impacted the students’ learning style differences’ in the **Abstract**.

2) In the original submission, we used the expression ‘a clearer picture’ due to the following two reasons. First, the data of our analysis was collected through a compulsory blended college English course where students across 46 disciplines were enrolled. Compared with previous studies, this investigation attempted to take into consideration more inclusive disciplines so that we can get a better understanding of students’ learning styles across different domains. Second, the SVM boasts powerful ability to detect the latent interactions between a large number of variables compared with other conventional statistic methods. However, due to our negligence, the comparative degree was not supported by any of the objects that we intended to compare our analysis with. In addition, we also believe that the use of comparative degree is not appropriate here because of the limitations that we have acknowledged in the **Conclusion** part. Thanks again to the reviewer for pointing out the pitfalls in our argument. During the revision, we have changed the use of comparative degree by using the original degree and further explaining the uniqueness of our study, making it easier for the readers to grasp the main idea.

3) Admittedly, the use of the word ‘omnipresent’ is inappropriate here. We intended to indicate that having been discussed in a series of scholarly works, academic discipline is an important variable concerning students’ learning styles. However, ‘omnipresent’ in fact means ‘being present everywhere at once’, which cannot be applied to this context. Thanks to the reviewer for reminding us of this inaccuracy. During the revision, we have changed the word ‘omnipresent’ into ‘important’ to clarify the argument.

The revised parts have been provided here in blue font for ease of reference.

(Lines 39-41) … The findings of this study shed light on the optimal feature sets that collectively determined students’ discipline-specific learning styles in a college blended learning setting.

(Lines 66-72) … Based on data for students from 46 disciplines, this research delves into the effects of a massive quantity of variables related to students’ learning styles with the help of a powerful machine learning algorithm. Considering the convergence of a wide range of academic disciplines and the detection of latent interactions between a large number of variables, this study aims to provide a clear picture of the relationship between disciplinary factors and students’ learning styles in a blended learning setting.

(Lines 126-128) … In the higher education context, the academic discipline seems to be an important variable that influences students’ distinctive learning styles, which echoes a multitude of investigations.

**Comment 5**

(2) Wordy sentences, such as ‘.. has always been a long-lasting and hot-debated topic….. (Line 48)’ and ‘the present study is designed to achieve two major objectives (Line 60)’. These two sentences might be re-wrote as ‘… has been constantly discussed’ and ‘this study aims to achieve …’.

**Response to comment 5**

Thanks to the reviewer for pointing out this problem and providing constructive suggestions for it. We feel sorry that in the original submission some wordy sentences have caused many troubles in reading. According to the suggestion, we have made the two sentences mentioned more precise and concise using ‘…has been constantly discussed’ and ‘this study aims to achieve …’. The revised parts have been marked in blue font and provided below for ease of reference:

(Lines 46-47) …Learning style, as an integral and vital part of a student’s learning process, has been constantly discussed in the field of education and pedagogy….

(Lines 58-59) …In an effort to address this gap, this study aims to achieve two major objectives….

**Comment 6**

(3) Hard-to-read long sentences, such as ‘First, …. 46 disciplines were enrolled (Line 60-63)’. The authors are suggested to re-organize the long sentences into a few logically connected short sentences, which are easy for the readers to follow.

**Response to comment 6**

Many thanks to the reviewer for this constructive suggestion. We felt very sorry that we did not pay attention to this tricky long sentence. According to the suggestion, we have cut this sentence into two, making it easier for the readers to follow. The revised part has been marked in blue font and also been provided here for the ease of reference:

(Lines 59-63) … First, it investigates how disciplinary background impacts students’ learning styles in a blended learning environment based on data collected in a compulsory college English course. Students across 46 disciplines were enrolled in this course, providing numerous disciplinary factor resources for investigating learning styles.

**Comment 7**

(5) The authors are suggested to use hedge words instead of the words with a strong tongue (e.g., potent, omnipresent, a constellation of)

**Response to comment 7**

Our heartfelt thanks go to the reviewer for this invaluable and helpful suggestion. Indeed, using hedge words rather than words with a strong tongue could express an opinion more cautiously, making the argument rigorous and compelling. We sincerely apologize for not paying much attention to the wording in the original submission. According to the suggestion, we have rewritten the sentences in a more careful way. The expression ‘are potent predicator’ has been changed into ‘are generally regarded as essential predictors’, and ‘is an omnipresent variable’ into ‘seems to be an important variable’. The sentence which includes the phrase ‘a constellation of’ has been removed. The revised parts have been marked blue in the original manuscript and have been provided below for ease of reference:

(Lines 174-176) Considering that academic disciplines and learning environment are generally regarded as essential predictors of students’ learning styles, some studies have also concentrated on the effects of academic discipline in a blended learning environment.

(Lines 126-128) … In the higher education context, the academic discipline seems to be an important variable that influences students’ distinctive learning styles, which echoes a multitude of investigations.

**Comment 8**

2. Literature review. (1) Why did the authors write ‘The examination of a constellation … and extended (Line 71-73)’ as the beginning of this paragraph? What is the connection between this sentence and the following contents?

**Response to comment 8**

We are grateful to the reviewer for pointing out this problem. Upon closer examination, we concur completely with the reviewer that the redundant sentence ‘The examination of a constellation … and extended (Lines 71-73)’ does not have any connection with the following contents. According to the valuable suggestion, we have deleted this sentence.

**Comment 9**

(2) Could the authors add some details about ‘analytical lenses (Line 88)’?

**Response to comment 9**

Thanks for offering us this opportunity to further clarify the major learning style models. In the original submission, we did not include a brief introduction to these well-established and widely-used learning style models, namely, the Kolb model, the Myers-Briggs Type Indicator model and the Felder-Silverman learning style model (FSLSM). This has led to an insufficient theoretical basis for this investigation. According to the suggestion, we have carefully reread the related scholarly works about the learning style models mentioned above. Focusing on the thinking process of learners, the Kolb model classifies students’ learning styles into four types; while the Myers-Briggs Type Indicator model mainly focuses on the learning resources of students, dividing learners into two types. The FSLSM, which was used in this study, identifies four paired dimensions that delicately distinguished students’ engagement in learning process. The basic descriptions of these analytical lenses have been added during revision and marked in blue font, which is also provided here for the ease of reference:

(Lines 84-104) In addition, a strong concern in this field during the last three decades has led to a proliferation of models that are germane to learning styles, including the Kolb model [18], the Myers-Briggs Type Indicator model [19] and the Felder-Silverman learning style model (FSLSM) [20]. These learning style models have provided useful analytical lenses for analyzing students’ learning styles. The Kolb model focuses on learners’ thinking processes and identifies four types of learning, namely, diverging, assimilating, converging, and accommodating [18]. The Myers-Briggs Type Indicator model classifies learners into extraversion and introversion types, with the former preferring to learn from interpersonal communication and the latter inclining to benefit from personal experience [19]. As the most popular available model, the FSLSM identifies eight categories of learners according to the four dimensions of perception, input, processing and understanding [20]. In contrast to other learning style models that divided students into only a few groups, the FSLSM describes students’ learning styles in a more detailed manner. The four paired dimensions delicately distinguish students’ engagement in the learning process, providing a solid basis for a steady and reliable learning style analysis [21]. In addition, it has been argued that the FSLSM is the most appropriate model for a technology-enhanced learning environment because it involves important theories of cognitive learning behaviors [22–23]. Therefore, a large number of scholars have based their investigations of students’ learning styles in the e-learning/computer-aided learning environment on FSLSM [24–28].

**Comment 10**

(3) It might not be appropriate to use the conjunction word ‘Despite (Line 88)’, because I could not figure out the adversative relation. I guess that the author may intend to state ‘FSLSM was adopted most’.

**Response to comment 10**

This important piece of advice is highly appreciated. We feel sorry that the use of the conjunction word ‘Despite (Line 88)’ has caused misunderstanding. According to the suggestion, we have deleted this word during the revision.

**Comment 11**

(4) I suggest that the author add a subheading (maybe: Learning style and FSLSM) before the second paragraph in the Literature review section to orient the readers.

**Response to comment 11**

The reviewer’s valuable suggestion is greatly appreciated. According to this suggestion, we have added a subheading of **Learning style and FSLSM** in the **Literature Review** section to orient the readers. Following this logic, we have also added a subheading of **Learning style and academic disciplines** to make the structure of the **Literature Review** section clearer and more organized. The revised structure of the literature review has now been provided below in blue font for the ease of reference:

**Literature review**

**Theories of learning styles**

(Line 105) **Learning styles and FSLSM**

(Line 123) **Learning styles and academic disciplines**

(Line 138) **Learning styles in an online/blended learning environment**

**Comment 12**

(5) The transition from the second paragraph to the third paragraph was not smooth; the authors are recommended to add one or two transitional sentences.

**Response to comment 12**

The reviewer’s suggestion is greatly appreciated. During revision, we have added appropriate transitional sentence to make the transition between different paragraphs smoother. The revised part has been marked in blue in the manuscript, which is also provided here for the ease of reference:

(Lines 56-57) Researchers in recent years have explored students’ learning styles from various perspectives [11-13]. However, knowledge of the learning styles of students from different disciplines in blended learning environments is limited. In an effort to address this gap, this study aims to achieve two major objectives…

**Comment 13**

(6) Any other reasons for the authors’ application of FSLSM?

**Response to comment 13**

Many thanks to the reviewer for this constructive suggestion. The FSLSM has been selected because of the following four reasons. First, the FSLSM has been utilized in a series of studies within the blended learning context (e.g., Abdullah, 2015; Azzi, Jeghal, Radouane, Yahyaouy, & Tairi, 2020; Garcia, Amandi, Schiaffino, & Campo, 2007; Hmedna, El Mezouary, & Baz O, 2020). Second, this comprehensive, well-established and widely-used model has been verified in previous studies (Crockett et al., 2011; Latham, Crockett, McLean, & Edmonds, 2012). Third, the FSLSM enjoys a good predictive ability with independent testing sets (Curry, 1987), and has been developed considering students interaction with the external learning environment. Fourth, the ILS is a free instrument that can be openly accessed online.

According to the suggestion, we have added the additional justification for using the FSLSM in our analysis. In the section of “Theories of learning styles”, we have compared the FSLSM with other popular learning style models. In addition, we have carefully delved into the related scholarly works, enumerating a series of studies on students’ behavior in the blended environment on the basis of this model. In addition, in the “Instrument” section, we have clearly stated the three reasons why we have selected this model. After this adjustment, the manuscript can not only better elaborate our initial thought on the choice of this appropriate model, but also be more constructive for related research on learning styles in the blended learning environment. The revised parts have been marked in blue font and also provided below for the ease of reference:

**Theories of learning styles**

(Lines 94-104) …As the most popular available model, the FSLSM identifies eight categories of learners according to the four dimensions of perception, input, processing and understanding [20]. In contrast to other learning style models that divided students into only a few groups, the FSLSM describes students’ learning styles in a more detailed manner. The four paired dimensions delicately distinguish students’ engagement in the learning process, providing a solid basis for a steady and reliable learning style analysis [21]. In addition, it has been argued that the FSLSM is the most appropriate model for a technology-enhanced learning environment because it involves important theories of cognitive learning behaviors [22–23]. Therefore, a large number of scholars have based their investigations of students’ learning styles in the e-learning/computer-aided learning environment on FSLSM [24–28].

**Instrument**

(Lines 309-330) In learning style research, it is difficult to select an instrument to measure the subjects’ learning styles [73]. The criteria used for the selection of a learning style instrument in this study include the following: 1) successful use of the instrument in previous studies, 2) demonstrated validity and reliability, 3) a match between the purpose of the instrument and the aim of this study and 4) open access to the questionnaire.

The Felder and Soloman’s ILS questionnaire, which was built based on the FSLSM, was adopted in the present study to investigate students’ learning styles across different disciplines. First, the FSLSM is recognized as the most commonly used model for measuring individual learning styles on a general scale [74] in higher education [75] and has remained popular for many years across different disciplines in university settings and beyond. In the age of personalized instruction, this model has breathed new life into areas such as blended learning [76], online distance learning [77], courseware design [78], and intelligent tutoring systems [79-80]. Second, the FSLSM is based on previous learning style models; the FSLSM integrates all their advantages and is, thus, more comprehensive in delineating students’ learning styles [81-82]. Third, the FSLSM has a good predictive ability with independent testing sets (i.e., unknown learning style objects) [17], which has been repeatedly proven to be a more accurate, reliable, and valid model than most other models for predicting students’ learning performance [10, 82]. Fourth, the ILS is a free instrument that can be openly accessed online (URL: https://www.webtools.ncsu.edu/learningstyles/) and has been widely used in the research context [83-84].

**Comment 14**

(7) The reviews on learning styles in an online/blended learning environment were not enough, the authors are suggested to add additional studies and summarize the main findings of them. Based on this, the authors could propose research gaps and questions.

**Response to comment 14**

We would like to thank the reviewer for pointing out the lack of literature from this aspect. During the revision, we provided a detailed section of literature review, which dealt with four related aspects, namely, theories of learning styles, learning styles and FSLSM, learning style and academic disciplines, and learning styles in an online/blended learning environment, respectively, respectively. In a nutshell, we felt sorry that we did not provide sufficient literature to support our assumption, and the corresponding modifications were highlighted in blue font in the revised manuscript and the whole picture of the literature review is provided below in blue font for the ease of reference:

**Learning styles in an online/blended learning environment**

(Lines 139-173) Individuals’ learning styles reflect their adaptive orientation to learning and are not fixed personality traits. Consequently, learning styles can vary among diverse contexts, and related research in different contexts is vital to understanding learning styles in greater depth. Web-based technologies eliminate barriers of space and time and have become integrated in individuals’ daily lives and learning habits. Online and blended learning have begun to pervade virtually every aspect of the education landscape [40], and this warrants close attention. In addition to a series of studies that reflected upon the application of information and communication technology in the learning process [45-46], recent studies have found a mixed picture of whether students in a web-based/blended learning environment have a typical preference for learning.

Online learning makes it possible for students to set their goals and develop an individualized study plan, equipping them with more learning autonomy [47]. Generally, students with a more independent learning style, greater self-regulating behavior and stronger self-efficacy are found to be more successful in an online environment [48]. For now, researchers have made substantial contributions to the identification and prediction of learning styles in an online learning environment [27, 49-52]. For instance, an inspiring study focused on the manifestation of college students’ learning styles in a purely computer-based learning environment to evaluate the different learning styles of web-learners in the online courses, indicating that students’ learning styles were significantly related to online participation [50]. Students’ learning styles in interactive E-learning have also been meticulously investigated, from which online tutorials have been found to be contributive to students’ academic performance regardless of their learning styles [52].

As a flexible learning method, blended courses have combined the advantages of both online learning and traditional teaching methods [53]. Researchers have investigated students’ learning styles within this context and have identified a series of prominent factors, including perceived satisfaction and technology acceptance [54], the dynamics of the online/face-to-face environment [55], and curriculum design [56]. Based on the Visual, Aural, Reading or Write and Kinesthetic model, a comprehensive study scrutinized the learning styles of K12 students in a blended learning environment, elucidating the effect of the relationship between personality, learning style and satisfaction on educational outcomes [57]. A recent study underscored the negative effects of kinesthetic learning style, whereas the positive effects of visual or auditory learning styles on students’ academic performance, were also marked in the context of blended learning [58].

**Comment 15**

3. Materials and methods. This part is organized with sufficient justification, but the authors should add some details. (1) Would it possible to draw a simple flow chart to describe the whole experiment process?

**Response to comment 15**

We are extremely appreciative of the reviewer’s detailed and professional suggestion, which give us a clear sense of what we missed and should do when revising the manuscript. The SVM has recently garnered massive popularity in academia and beyond due to its high performance in precisely recognizing underlying patterns from a myriad of variables precisely without strong preconceptions. However, its complex mechanism warrants elaboration for a better understanding of its functions. In our experiment, we first collected data based on the ILS in the compulsory English courses, after asking for the permission of both the students and the students. Second, according to Biglan’s taxonomy of academic disciplines, we classified students into four groups, namely, HA, HP, SA and SP. Third, during data preprocessing, we used the median to impute the missing data, and standardized the data between 0 to 1. Fourth, the SPSS ANOVA was used to analyze the data. Fifth, we coded students from four different disciplines and put the data into the machine to run the program. The SVM(-RFE) then produced the final results, which include the indicators of performance and the ranking of the variables. The process was then repeated to get multiple comparisons between different disciplines. In addition, we have selected the top 20 features according to the previous research and the indicators. In the revised manuscript, we have added a simple flow chart which could give the readers. Readers will be directed to this chart by a sentence “The experiment process is provided below in Fig 3 for the ease of reference.” The revised part is provided below in blue font for the ease of reference:

(Lines 485-486) …The experimental process is provided below in Fig 3 for the ease of reference.

Fig 3. Experiment process and working mechanism of SVM and SVM-RFE.

**Comment 16**

(2) For the first time to use ‘ILS’, please write the full name.

**Response to comment 16**

Thanks to the reviewer for pointing out the imprecision in our arguments. We felt sorry that we did not notice this mistake in the original manuscript. According to the suggestion, we have provided the full name of the ILS, namely, Inventory of Learning Styles when it first appeared. In addition, we have carefully checked the whole manuscript in order to avoid making this kind of mistakes again. The revised part has been marked blue in the original manuscript and also provided below for the ease of reference:

(Line 133-135) In particular, using the Inventory of Learning Styles (ILS) questionnaire and found prominent disparities in learning styles between students from four different disciplinary backgrounds in the special educational field of vocational training [44].

**Comment 17**

4. Results. (1) It is a bit hard to follow the research results described by the authors, would it possible to generally summarize the main results in a table?

**Response to comment 17**

Many thanks to the reviewer for this constructive comment. In order to make the research results more concise but informative enough, we have combined the previous three tables in the results section into one. This newly-developed table includes not only the indicators of the original SVM models and the models composed of 20 variables, which was produced by the SVM algorithm, but also the ranking of the 20 variables for each model produced by the SVM-RFE. The newly-added is marked blue in the original manuscript and provided here for the ease of reference:

**Table 2. Results produced by SVM and SVM-RFE.**

| **Model** | **Algorithm** | **ACC** | **SPE** | **SEN** | **AUC** | **F-measure** | **Top 20 features** |
| --- | --- | --- | --- | --- | --- | --- | --- |
| **H-S** | SVM | 66.67% | 69.32% | 63.38% | 66.35% | 66.67% | 1, 2, 4, 5, 7, 8, 9, 10, 11, 14, 21, 22, 23, 26, 27, 29, 31, 32, 35, 39 |
|  | SVM-RFE | 87.30% | 88.18% | 86.20% | 87.19% | 87.30% |  |
| **A-P** | SVM | 64.52% | 77.78% | 55.08% | 64.93% | 64.52% | 3, 4, 5, 6, 7, 11, 15, 17, 18, 19, 21, 30, 31, 32, 33, 35, 39, 42, 43, 44 |
|  | SVM-RFE | 73.44% | 86.55% | 79.28% | 75.09% | 74.44% |  |
| **HA-HP** | SVM | 69.32% | 80.00% | 67.95% | 73.97% | 69.32% | 1, 4, 5, 6, 7, 8, 10, 12, 13, 14, 19, 21, 26, 28, 29, 31, 34, 39, 40, 44 |
|  | SVM-RFE | 82.59% | 82.30% | 78.26% | 89.13% | 79.59% |  |
| **HA-SA** | SVM | 68.47% | 63.64% | 70.51% | 67.07% | 68.47% | 1, 2, 4, 7, 8, 9, 10, 11, 14, 18, 21, 26, 27, 29, 32, 35, 37, 38, 39, 41 |
|  | SVM-RFE | 77.74% | 75.00% | 68.22% | 78.11% | 77.74% |  |
| **HA-SP** | SVM | 68.97% | 73.68% | 66.67% | 70.18% | 68.97% | 1, 2, 7, 9, 10, 11, 12, 14, 19, 20, 21, 22, 23, 27, 31, 32, 34, 35, 37, 39 |
|  | SVM-RFE | 76.09% | 74.00% | 67.70% | 77.35% | 69.09% |  |
| **HP-SA** | SVM | 55.16% | 56.52% | 58.00% | 57.76% | 56.16% | 1, 2, 4, 5, 6, 7, 9, 10, 12, 15, 16, 21, 26, 27, 28, 29, 31, 33, 39, 44 |
|  | SVM-RFE | 74.63% | 72.00% | 70.04% | 75.02% | 69.63% |  |
| **HP-SP** | SVM | 58.33% | 57.90% | 60.00% | 58.95% | 58.33% | 1, 2, 5, 7, 8, 12, 13, 15, 16, 18, 19, 21, 23, 27, 29, 32, 33, 34, 35, 40 |
|  | SVM-RFE | 70.83% | 75.00% | 68.00% | 72.50% | 70.83% |  |
| **SA-SP** | SVM | 60.00% | 63.16% | 56.25% | 59.70% | 60.00% | 3, 4, 5, 7, 8, 9, 11, 13, 15, 18, 19, 21, 27, 31, 32, 35, 37, 39, 41, 44 |
|  | SVM-RFE | 70.46% | 75.00% | 76.25% | 71.28% | 71.46% |  |

*Note*: Indicators in the upper row belong to original SVM models, while those in the lower row belong to the models with 20 features.

**Comment 18**

(2) Why did the authors choose 20 features?

**Response to comment 18**

Thanks to the reviewer for providing us with opportunities to elaborate the reasons why we have selected 20 features. The two reasons are data-based and literature-based, respectively. On the one hand, it is clear that indicators of models composed of 20 features have a better performance than the original models. Once the features outnumber 20, the indicators of the models will decrease, which means that the performance of the model could be negatively influenced. On the other hand, based on previous SVM-based studies in the social sciences, 20 to 30 features were identified as a good number for an optimal feature set (Mou et al., 2014). The revised part is marked blue in the original manuscript and provided here for the ease of reference:

**Results**

(Lines 489-500) The classification results produced by SVM and the ranking of the top 20 features produced by SVM-RFE were listed in Table 2. Twenty variables have been selected in this study for two reasons: a data-based reason and a literature-based reason. First, it is clear that models composed of 20 features generally have a better performance than the original models. The performance of models with more than 20 is negatively influenced. Second, SVM-based studies in the social sciences have identified 20 to 30 features as a good number for an optimal feature set [110], and 20 features were selected for inclusion in the optimal feature set [97]. Therefore, in this study, the top 20 features were selected for subsequent analysis, as proposed in previous analyses that yielded accepted measurement rates. These 20 features retained most of the useful information from all 44 factors but with fewer feature numbers, which showed satisfactory representation [98].

**Comment 19**

(3) The authors are recommended to present the results as per the proposed questions. For example, subheading ‘Results of question one’.

**Response to comment 19**

Thanks to the reviewer for giving us this invaluable suggestion, which helps us better structure our results section. According to the suggestion, we have re-organized the sub-title of our **Results** section, making it compatible with the **Discussion** section. The revised Results section is now organized according to the three research questions we proposed. The content of each sub-section has also been adjusted. The revised parts have been marked blue in the original manuscript and provided here for the ease of reference:

**Results**

(Lines 503-505) **Results of RQ (1) What are the differentiability levels among different academic disciplines based on students’ learning styles?**

…

(Lines 547-549) **Results of RQ (2) What are the key features that can be selected to determine the collective impact on differentiation by a machine learning algorithm?**

…

(Lines 578-604) **Results of RQ (3) What are the collective impacts of optimal feature sets?**

The collective impacts of optimal feature sets could be interpreted from four aspects, namely, the complexities of students’ learning styles, the appropriate choice of SVM, the ranking of SVM-RFE and multiple detailed comparisons between students from different disciplines. First, the FSLSM considers the fact that students’ learning styles are shaped by a series of factors during the growth process, which intertwine and interact with each other. Considering the complex dynamics of the learning style, selecting an approach that could detect the combined effects of a group of variables is needed. Second, recent years have witnessed the emergence of data mining approaches to explore students learning styles [28,49-51,112]. Specifically, as one of the top machine learning algorithms, the SVM excels in identifying the combined effects of high-order factors [89]. In this study, the SVM has proven to perform well in classifying students’ learning styles across different disciplines, with every indicator being acceptable. Third, the combination of SVM with RFE could enable the simultaneous discovery of multiple features that collectively determine classification. Notably, although SVM-FRE could rank the importance of the features, they should be regarded as an entire optimal feature set. In other words, the combination of these 20 features, rather than a single factor, could differentiate students’ learning styles across different academic disciplines. Last but not least, the multiple comparisons between different SVM models of discipline provide the most effective learning style factors, giving researchers clues to the nuanced differences between students’ learning styles. It can be seen that students from different academic disciplines understand, see and reflect things from individualized perspectives. The 20 most effective factors for all models scattered within 1 to 44, verifying students’ different learning styles in 4 dimensions. Therefore, the FSLSM provides a useful and effective tool for evaluating students’ learning styles from a rather comprehensive point of view.

**Comment 20**

5. Discussion. Try to write with a general-specific structure. Writing a summative sentence at the beginning of each paragraph to present the core idea, then explain it.

**Response to comment 20**

The reviewer’s suggestion has been greatly appreciated. During revision, we have added a summative sentence at the beginning of each paragraph to present the core idea in each sub-section in the **Discussion** section. The revised parts have been marked blue in the original manuscript and provided here for the ease of reference:

**Levels of differentiability among various academic disciplines based on students’ learning styles with SVM**

(Lines 610-612) The results suggest that SVM is an effective approach for classification in the blended learning context in which students with diverse disciplinary backgrounds can be distinguished from each other according to their learning styles…

**Verification of the features included in the optimal feature sets**

(Lines 675-677) Features included in the optimal feature sets provided mixed findings compared with previous studies. Some of the 20 identified features are verified and consistent with previous studies…

**Interpretation of the collective impact of optimal feature sets**

(Lines 747-749) In each SVM model based on a two-discipline model, the 20 key features (collectively known as an optimal feature set) selected exert a concerted effect on students’ learning styles across different disciplines (see Table 2)…

**Comment 21**

6. The authors had better avoid using one-sentence paragraphs in an academic article.

**Response to comment 21**

Many thanks to the reviewer for this reminder. According to the suggestion, we have revised all of the one-sentence paragraphs in the original manuscript to make it comply with the academic norm.

**Comment 22**

7. The language may be more concise and information-intensive. For example, phrases and sentences like “In the field of learning style research, one great difficulty is the selection of proper instruments to measure the subjects’ learning styles [59].” and “Among the various inventories of learning styles” may be deleted.

**Response to comment 22**

Thanks to the reviewer for pointing out the redundancy of the language in the original manuscript. During revision, we have now carefully re-read the manuscript and removed re-written sentences like “In the field of learning style research, one great difficulty is the selection of proper instruments to measure the subjects’ learning styles” and deleted the expression “Among the various inventories of learning styles”. The revised parts have been marked blue in the original manuscript and provided here for the ease of reference:

(Lines 309-310) In learning style research, it is difficult to select an instrument to measure the subjects’ learning styles [73]…

(Lines 315-317) The Felder and Soloman’s ILS questionnaire, which was built based on the FSLSM, was adopted in the present study to investigate students’ learning styles across different disciplines…

**Comment 23**

8. It is observed that some sections begin with “First.” I suggest the authors add a topic sentence before “First,” briefly illustrating what will be presented. The titles could not replace topic sentences.

**Response to comment 23**

Thanks to the reviewer for pointing out this drawback in our argument. In view of the **Comment 20**, we have added a summative sentence at the beginning of each paragraph to present the core idea in each sub-section in the Discussion section, avoiding the use of “First” at the beginning of each paragraph. The revised parts have been marked blue in the original manuscript and provided here for the ease of reference:

**Levels of differentiability among various academic disciplines based on students’ learning styles with SVM**

(Lines 610-612) The results suggest that SVM is an effective approach for classification in the blended learning context in which students with diverse disciplinary backgrounds can be distinguished from each other according to their learning styles…

**Verification of the features included in the optimal feature sets**

(Lines 675-677) Features included in the optimal feature sets provided mixed findings compared with previous studies. Some of the 20 identified features are verified and consistent with previous studies…

**Interpretation of the collective impact of optimal feature sets**

(Lines 747-749) In each SVM model based on a two-discipline model, the 20 key features (collectively known as an optimal feature set) selected exert a concerted effect on students’ learning styles across different disciplines (see Table 2)…

**Comment 24**

9. Based on the in-depth discussions and fruitful results, the authors may propose some implications for future researchers and educators.

**Response to comment 24**

We are grateful to the reviewer for this constructive comment to inspire researchers and us to carry on future work. During the revision, we revised the **Conclusion** section to draw suggestions for further research on students’ learning styles across disciplines in a blended learning environment. The revised part was provided as follows in blue font for ease of references:

(Lines 800-814) In conclusion, the findings of this research validate previous findings and offer new perspectives on students’ learning styles in a blended learning environment, which provides future implications for educational researchers, policy makers and educational practitioners (i.e., teachers and students). For educational researchers, this study not only highlights the merits of using machine learning algorithms to explore students’ learning styles but also provides valuable information on the delicate interactions between blended learning, academic disciplines and learning styles. For policy makers, this analysis provides evidence for a more inclusive but personalized educational policy. For instance, in addition to learning styles, the linkage among students’ education in different phases should be considered. For educational practitioners, this study plays a positive role in promoting student-centered and tailor-made teaching. The findings of this study can help learners of different disciplines develop a more profound understanding of their blended learning tendencies and assist teachers in determining how to bring students’ learning styles into full play pedagogically, especially in interdisciplinary courses [135-138].
